# The specific hallmarks, emerging roles, key mechanisms, and clinical applications of intra-tumoral microbiota in human cancers

**DOI:** 10.1016/j.gendis.2025.101733

**Published:** 2025-06-22

**Authors:** Tingting Zhao, Na Sun, Jun Ding, Zaihui Peng, Fei Han, Xiaowei Qi

**Affiliations:** a Department of Breast and Thyroid Surgery, Southwest Hospital, Army Medical University, Chongqing 400038, China; b Key Laboratory of Minimally Invasive Surgery and Precision Treatment for Breast Cancer of Chongqing Municipal Health Commission, Chongqing 400038, China; c Department of Hepatobiliary Surgery, Third Affiliated Hospital of Chongqing Medical University, Chongqing 401120, China; d Department of Toxicology, School of Public Health, Chongqing Medical University, Chongqing 400016, China; e Laboratory of Reproductive Biology, Chongqing Medical University, Chongqing 400016, China

**Keywords:** Cancer, Characteristics, Diagnosis, Intra-tumoral microbiota, Prognosis, Therapy

## Abstract

Intra-tumoral microbes have been revealed to exist in many cancer types, attracting widespread attention. The significance of intra-tumoral microbes is becoming increasingly apparent in various aspects of human cancers, encompassing cancer initiation, progression, metastasis, diagnostic approaches, prognostic evaluations, and therapeutic interventions. Despite the considerable focus dedicated to this topic by numerous scholars, a comprehensive analysis of intra-tumoral microbiota is still lacking in human cancers. Especially, identifying specific microbial hallmarks in the occurrence and development of cancer and different cancers remains the central task for investigators. This review focuses on the identification and analysis of distinct attributes and noteworthy characteristics exhibited by intra-tumoral microbiota across various types of cancer. The potential mechanisms of intra-tumoral microbiota action, as well as the significance of the microbiome in the diagnosis and prognosis of cancer, are systematically summarized. The capacity of intra-tumoral microbes to regulate cancer treatment with a focus on the relevant microbial species, and the possibility of targeting the microbiota to improve treatment effectiveness while preventing toxicity, are specifically highlighted. Lastly, the challenges, limitations, and prospects of intra-tumoral microbes in further study and clinical application, including prognostic, diagnostic, and therapeutic applications, are discussed in cancers. This review provides a systematic summary of the specific characteristics, molecular mechanisms, therapeutic effects, and diagnostic and prognostic values of intra-tumoral microbiota in different cancers, which will help improve the diagnosis, treatment, and prognosis of tumor patients and offer new ideas for achieving precise treatment of cancer with intra-tumoral microbiota.

## Introduction

The human microbiota consists of all microorganisms living in different places in the human body, including the oral cavity, respiratory tract, reproductive tract, and skin.^1^ Numerous studies have reported that human microbes are closely related to human diseases such as obesity, diabetes, hypertension, and malignant tumors.^2^, ^3^, ^4^ Recently, increased interest has been raised in the interaction between microbes and tumors. According to statistics in 2018, about 13% of global cancer incidence is attributable to microorganisms.^5^ Microorganisms exist in tumor tissues (intra-tumoral), and tissues adjacent to and/or distant from tumors (extra-tumoral), such as the digestive tract (gut), genitourinary tract, and respiratory tract.^6^ For a long time, gut microbiota has been the most widely followed and studied. Recently, the discovery of intra-tumoral microbiota has aroused the interest of researchers.^7^ The organs and tissues traditionally considered sterile are found to have different types of microorganisms, and especially tumor tissues have been shown to contain a large number of microorganisms. It has been reported that the intra-tumoral microbiota exists in a variety of major cancers, such as breast cancer, pancreatic cancer, lung cancer, prostate cancer, colorectal cancer (CRC), and ovarian cancer.^8^, ^9^, ^10^, ^11^ An increasing number of studies exhibit a close association of intra-tumor microbiota with tumor types, occurrence and development, immune regulation, and therapy.^12^^,^^13^ However, the characteristics, roles, and mechanisms, as well as the diagnostic, prognostic, and therapeutic potential of intra-tumoral microbiota in different tumors, are still not explored and determined due to scattered information reported. The purpose of this review is to attempt to address the issue by integrating and summarizing existing literature results to obtain valuable information on intra-tumoral microbiota in clinical applications.

### The intra-tumoral microbiota and human cancers

As early as 100 years ago, scientists discovered the presence of bacteria in human tumor tissue samples for the first time. With the advance of sequencing technology, the focus on the microbiome of tumor tissue is increasing. Microorganisms have been reported to exist in a variety of tumor tissues, such as gastric cancer, breast cancer, pancreatic cancer, lung cancer, prostate cancer, CRC, and ovarian cancer.^8^^,^^14^, ^15^, ^16^, ^17^, ^18^, ^19^ Pathological examinations have shown that the intra-tumor microorganisms are present mostly intracellular, both in cancer and immune cells.^8^

## Intra-tumoral microbiota and breast cancer

Breast tissue was once thought to be sterile, whereas the presence of bacteria in breast tumor cells has been confirmed. Breast cancers have a richer and more diverse bacterial microbiome than other cancer types, and the bacterial load and richness are higher in breast cancer tissues than in normal samples.^8^ The most abundant phyla are *Proteobacteria*, *Actinobacteria*, *Firmicutes*, and *Bacteroidetes* in breast tissue.^20^ It has been shown that no differences in bacterial types are observed between cancer tissues and adjacent normal tissues in breast cancer patients by 16S rRNA amplicon sequencing. However, compared with normal tissue of healthy controls, higher abundances of *Staphylococcus*, *Enterobacteriaceae*, and *Bacillus* are found in tumor tissues of breast cancer; on the contrary, higher abundances of *Streptococcus* and *Lactococcus* are observed in healthy tissues than breast cancer tissues.^8^^,^^15^^,^^21^ Breast malignancy is associated with the enrichment of lower-abundance bacterial taxa, and the bacterial taxa include *Lactobacillus*, *Fusobacterium*, *Hydrogenophaga*, *Atopobium*, and *Gluconacetobacter*.^22^ Moreover, the relative abundance of family *Bacteroidaceae* is decreased, and the abundance of the genus *Agrococcus* is increased with the development of breast cancer.^23^ Bacterial microbiome signatures differ between breast cancer subtypes.^24^^,^^25^ *Actinomyces*, *Brevundimonas*, *Bartonella*, *Coxiella*, *Sphingomonas*, *Mycobacterium*, and *Mobiluncus* can be detected in the samples of all breast cancer subtypes.^24^ The bacterial signature in the estrogen receptor (ER) subtype is the most complex. *Actinomycetaceae* family, *Sphingomonas_US124*, *Streptophyta_UF_UG116* genus, *Alkanindiges* genus, *Actinomyces odontolyticus*, and *Lautropia_US38* are enriched in ER^−^ breast cancer, while *Arcanobacterium*, *Bifidobacterium*, *Cardiobacterium*, *Citrobacter*, *Escherichia*, and *Bartonella* genus are significantly detected in ER ^+^ breast cancer samples.^8^^,^^24^ *Granulicatella_US31*, *Streptococcus*, and *Dyadobacter* genera are enriched in human epidermal growth factor receptor 2-positive (HER2^+^) breast cancer.^8^^,^^24^ *Bordetella*, *Campylobacter*, *Legionella*, and *Pasteurella* are significantly enriched in triple-positive breast cancer (TPBC).^15^^,^^24^ *Aerococcus*, *Arcobacter*, *Geobacillus*, *Orientia*, *Rothia*, *Achromobacter denitrificans*, *Leptotrichia_US21*, *Bacillus_US21*, *Streptophyta_UF_UG116*, *Turicibacter* genus, *Nocardiopsaceae* family, and *Achromobacter* genus are enriched, while *Actinomyces* is markedly low in triple-negative breast cancer (TNBC).^15^^,^^24^^,^^26^ The results of the above research suggest that the various subtypes of breast cancer have varied bacterial micro-organisms with aspects that are specific to each type as well as shared components, indicating that the changes in composition and function of bacteria may be driving breast cancer progression. Besides, there are geographic differences in the bacterial microbiome in breast cancer.^27^^,^^28^ *Staphylococcus* and *Enterobacteriaceae* in normal breast tissue are more abundant in Irish samples than in Canadian samples.^27^ In Asiatic patients, an increased representation of the *Propionicimonas* genus and *Micrococcaceae*, *Methylobacteriaceae*, *Caulobacteraceae*, *Nocardioidaceae*, and *Rhodobacteraceae* families has been observed in breast cancer tissues.^23^ In Canadian patients with TNBC, a higher abundance of *Enterobacteriaceae*, *Bacillus*, and *Staphylococcus* in breast cancer tissue has been found compared with normal breast tissue.^28^ These differences may be associated with different regions' eating habits, living environments, and metabolic levels.^29^

The predominant fungal, viral, and parasitic signatures are detected in breast cancer tissues. The fungi of *Ajellomyces*, *Rhizomucor*, *Alternaria*, *Filobasidiella*, *Cunninghamella*, *Trichophyton*, and *Epidermophyton* are detected in breast cancer samples, while not detected in healthy samples.^24^ The fungi of *Filobasidiella*, *Trichophyton*, and *Mucor* have been found to specifically present in ER ^+^ breast cancer samples, *Epidermophyton*, *Fonsecaea*, and *Pseudallescheria* uniquely exist in HER2^+^ breast cancer samples, *Penicillium* specifically presents in TPBC samples, and *Alternaria*, *Malassezia*, *Piedraia*, and *Rhizomucor* uniquely exist in TNBC samples.^24^ Moreover, *Fonsecaea pedrosoi*, *Piedraia hortae*, *Phialophora verrucosa*, *Paecilomyces reniformis*, and *Pleistophora mulleris* are detected in most TNBC tissues, while they are underrepresented in normal samples.^25^ It is of note that characteristic changes have been observed in the virome of breast cancer tissues. The viral families, including *Adenoviridae*, *Filoviridae*, *Anelloviridae*, *Bunyaviridae*, *Arenaviridae*, *Flaviviridae*, *Coronaviridae*, *Iridoviridae*, *Herpesviridae*, *Paramyxoviridae*, *Papillomaviridae*, *Picornaviridae*, *Parvoviridae*, *Reoviridae*, *Poxviridae*, *Rhabdoviridae*, and *Retroviridae*, are detected in the samples of all the breast cancer types compared with the control group.^24^ Some viral families are detected significantly only in a specific subtype of breast cancer. *Birneviridae* and *Hepeviridae* families are only detected in TPBC samples, and *Nodaviridae* is only detected in HER2^+^ breast cancer samples.^24^ Furthermore, *Herpesviridae* (HCMV, HHV1, KSHV, and EBV/HHV4), *Retroviridae* (Fujinami sarcoma virus and mouse mammary tumor virus (MMTV)), *Parapoxviridae*, *Polyomaviridae* (Merkel cell polyomavirus and simian virus 40), *Papillomaviridae* (HPV6b, HPV18, HPV2, and HPV16) families are detected in TNBC samples.^25^ As in the case of fungi, no single genus of parasite is detected in all four breast tumor subtypes.^24^ *Paragonimus* and *Brugia* are only detected in ER^+^ breast cancer samples, *Balamuthia* is positively correlated with HER2^+^ breast cancer samples, *Ancylostoma*, *Echinococcus*, *Angiostrongylus*, *Trichomonas*, *Sarcocystis*, and *Trichostrongylus* are found to be uniquely positively correlated with TPBC samples, and *Leishmania*, *Centrocestus*, *Necator*, *Contracaecum*, *Toxocara*, *Onchocerca*, *Trichuris*, and *Trichinella* are significantly detected only in TNBC samples.^24^ In addition, *Mansonella* and *Strongyloides* are also detected in TNBC samples, and *Trichuris trichura*, *Thelazia gulosa*, and *Leishmania* are the major compositions in TNBC samples.^25^ Up to now, *Archaea* have not been found in normal breast tissue and breast cancer tissue samples. These data show that intra-tumoral microbiota has robust viral, fungal, and parasitic signatures, and obvious differences in viral, fungal, and parasitic signatures exist in different subtypes of breast cancer.

## Intra-tumoral microbiota and lung cancer

Growing research has shown that lung tissues are not sterile but harbor complex, diverse communities of microbes.^30^^,^^31^ At present, there are more microbiological studies on oral and sputum samples of lung cancer patients, while fewer studies are reported on characterizing microbiomes in lung cancer tissues. A recent study has shown a distinct lung bacterial microbiome in lung tumor patients by examining the presence of lung tissue microbiome.^31^ At the phylum level, *Proteobacteria* is increased and *Firmicutes* is decreased in lung cancer tissues compared with non-cancer tissues.^31^ Lower bacterial diversity has also been found in lung tumor tissues compared with non-tumor tissues.^30^ Moreover, the genus *Thermus* is more abundant in lung tumor tissues from advanced-stage patients (IIIB and IV), and *Legionella* is higher in the tissues of lung cancer patients who developed metastases.^30^ Similarly, another study also shows a significant decrease in bacterial diversity in lung cancer tissues compared with normal controls.^32^ Genus *Streptococcus* is significantly more abundant in cancer cases than in the controls, while *Staphylococcus* is more abundant in the controls.^32^ Interestingly, *Dialister* and *Staphylococcus* abundance peaked in healthy samples, gradually declined in non-cancerous tissues of lung tumor patients, and reached the lowest presence in tumor tissues, whereas *Streptococcus* and *Neisseria* significantly increased in the tissues from healthy samples to lung cancer samples.^32^ Moreover, the bacterial abundance of families *Lachnospiraceae*, *Ruminococcaceae*, and *Bacteroidaceae*, and genera *Bacteroides*, *Ruminococcus*, *Roseburia*, and *Faecalibacterium* are significantly enriched in normal lung tissues, while the abundance of *Sphingomonadaceae* and *Koribacteraceae* is significantly enriched in lung cancer tissues.^33^ Compared with adjacent non-tumor tissues, lung cancer samples show lower community richness (α diversity), whereas no difference in overall bacterial microbiome dissimilarity (β diversity), and at the taxa level, the *Propionibacterium genus* is reduced significantly in lung cancer tissues compared with adjacent non-cancer tissues.^34^ Usually, each patient with non-small cell lung cancer seems to possess its bacterial signature, and its two main subtypes, adenocarcinoma and squamous cell carcinoma tissue, have no distinct bacterial profiles that are shared by every patient.^35^ Furthermore, the enteric, pathogenic, and pro-inflammatory bacteria, such as *Faecalibacterium*, *Escherichia-Shigella*, *Alloprevotella*, *Brevundimonas*, and *Pseudomonas*, are more frequently found in non-small cell lung cancer than healthy lung tissues.^35^ A complete genome of trichodysplasia spinulosa-associated polyomavirus has been determined from lung adenocarcinoma tissue samples.^36^ However, it seems that fungi and parasites still have not been reported in lung cancer tissues at present.

## Intra-tumoral microbiota and prostate cancer

Analyses of prokaryotic and viral DNA sequences indicate the presence of multiple and diverse microorganisms, including bacterial, viral, parasitic, and fungal in prostate cancer tissues.^37^^,^^38^ The microbiome in prostate tumor tissue is quite diverse when compared with that in normal tissue, which may provide markers for diagnostic and prognostic purposes in prostate tumors.^37^^,^^38^ As early as 2000, bacterial DNA sequences were found, and subsequently, the bacteria have indeed been detected by multiple studies in prostate cancer tissues.^24^^,^^39^, ^40^, ^41^, ^42^ The bacteria isolated are mostly gram-negative with the phyla in descending magnitude of *Proteobacteria*, *Actinobacteria*, *Bacteroidetes*, and *Firmicutes*, and among these, *Proteobacteria* is the most predominant phylum in prostate cancer tissues.^24^ Significant differences are observed in specific bacterial populations among tumor and non-tumor prostate specimens at certain genera levels. *Propionibacterium* spp. is the most abundant, and *Staphylococcus* spp. is more represented in prostate tumor tissues.^43^ The bacteria at the genus level, such as *Pseudomonas*, *Escherichia*, *Acinetobacter*, and *Propionibacterium*, are enriched in prostate cancer tissues.^44^ In particular, *Propionibacterium acnes* has been implicated in the development of prostate cancer.^40^ *Helicobacter pylori* (*H. pylori*) has been detected in prostate cancer tissues, and *H. pylori* may integrate the cagA sequences into specific chromosomes of tumor cells in prostate cancer.^24^^,^^45^ Some known and potential tumorigenic viruses have been identified in prostate cancer tissues, such as high-risk HPV strains 16 and 18, human cytomegalovirus (HCMV), and Epstein–Barr virus (EBV).^24^^,^^46^ Pieces of evidence have shown that BK polyomavirus (BKV) and JC polyomavirus (JCV) are more commonly detected in prostate cancer tissues than in normal controls, and BKV may exert its oncogenic activity at early stages of prostate cancer.^47^^,^^48^ Other endogenous retroviruses, such as gammaretrovirus MMLV, betaretrovirus MMTV, and alpharetrovirus RSV, have also been observed in prostate cancer tissues.^24^ The majority of fungi are *Ascomycota*, and the most prevalent fungal families detected are *dermatophytes*, *yeasts*, *zygomycetes*, and *microsporidia* in prostate tumor tissues.^24^ The parasitic signatures found in prostate cancer tissues have shown that *Nematoda* is the most prevalent, followed by *Platyhelminthes*, *Sarcomastigophora*, *Acanthocephala*, and *Apicomplexa*.^24^ *Chlamydia trachomatis* has been found in at least 90 % of prostate cancer tissue samples examined and is associated with an increased risk for the development of prostate cancer.^24^ In addition, *Mycoplasma genitalium* in tissues is independently associated with prostate cancer and with higher stages of prostate cancer patients.^49^ At present, archaea have still not been found in normal and prostate cancer tissue samples.

## Intra-tumoral microbiota and pancreatic cancer

Pancreatic cancer is a devastating and lethal malignancy, and abundant intra-tumoral microbes have been found in pancreatic cancer tissues.^17^^,^^50^^,^^51^ The number of *Bifidobacteria*, *Gammaproteobacteria*, *H. pylori*, and *Clostridium bacteria* in pancreatic cancer tissues showed a significant increase.^52^ In addition to *Porphyromonas gingivalis* (*P. gingivalis*) and *H. pylori*, high proportions of *Proteobacteria*, *Bacteroidetes*, and *Firmicutes* species are also found in pancreatic cancer tissues.^17^ Interestingly, *Fusobacterium* in pancreatic tumor tissues is related to worse prognosis of pancreatic cancer patients independently, indicating *Fusobacterium* may serve as a prognostic marker for pancreatic cancer.^53^ Unexpectedly, intra-tumoral bacteria are not always harmful to pancreatic cancer, and some bacteria are related to improved clinical outcomes. Alpha-diversity of tumor bacterial microbiome, defined as the number of species present within each tumor sample, is significantly higher in the long-term survivors compared with short-term survivors.^54^ Three intra-tumoral bacteria, *Sachharopolyspora*, *Pseudoxanthomonas*, and *Streptomyces*, are enriched in long-term survivors of pancreatic cancer patients, being predictive of long-term survival.^54^ In addition, *Bacillus clausii*, as one of the top species enriched in long-term survivors, can combine with the three genus signatures, *Sachharopolyspora*, *Pseudoxanthomonas*, and *Streptomyces*, to highly predict the long-term survival of pancreatic cancer patients.^54^ It has been revealed that HBV DNA can be detected in pancreatic cancer tissues, suggesting HBV infection in the pancreas may play an etiologic role.^55^ A higher abundance and distinct composition of fungi are detected in pancreatic tumor tissues compared with normal tissues, and *Malassezia* is enriched in pancreatic cancer tissues.^56^ Interestingly, microbiome dysbiosis may drive pancreatic carcinogenesis by modulating inflammatory processes. Some oral, gastric, and duodenal microbes can also colonize the pancreas and contribute to the development of pancreatic cancer.^57^ Up to now, archaea and parasites have not been found in pancreatic cancer tissues.

## Intra-tumoral microbiota and gastric cancer

Since *H. pylori* was reported in 1983, researchers have gradually realized the important role of microorganisms in the occurrence and development of gastric cancer.^58^ It has been widely accepted that chronic inflammation caused by *H. pylori* infection is closely related to precancerous lesions. Nevertheless, not all patients with *H. pylori* infection will develop gastric cancer, and eradication of *H. pylori* cannot completely prevent gastric cancer, suggesting that other microbiota in gastric tissues possibly play roles in the development of gastric cancer and maintenance of local lesion microenvironment. Recent studies have shown that the abundance and diversity of bacteria in gastric cancer tissues have increased, such as *Streptococcus*, *Peptostreptococcus*, and *Fusobacterium* being more abundant in tumor tissues than in adjacent non-tumor tissues.^59^, ^60^, ^61^, ^62^ The gastric microbiota is dominated by *Proteobacteria* in non-cancerous tissues and cancerous tissue samples, while the ranking of phyla slightly differed with an increased abundance of *Firmicutes*, *Fusobacteria*, *Bacteroidetes*, *Acidobacteria*, and *Actinobacteria*, but a decreased abundance of *Proteobacteria* in cancerous tissues when compared with non-cancerous tissues.^59^ The bacterial taxa enriched in gastric cancer tissues are predominantly represented by *Fusobacterium* and *Streptococcus*, whereas lactic acid-producing bacteria such as *Lactobacillus brevis* and *Lactococcus lactis* are more abundant in adjacent non-cancer tissues.^59^ Importantly, the abundance of *H. pylori* decreased in gastric cancer samples compared with non-cancerous tissues.^59^^,^^61^ Gastric mucosa analyses have shown a complex bacterial community in gastric cancer patients, and *Streptococcus*, *Lactobacillus*, *Veillonella*, and *Prevotella* are dominant.^63^ *Bacteroides uniformis* and *Prevotella copri* are significantly decreased, while *S. anginosus*, *Propionibacterium acnes*, and *Prevotella melaninogenica* are increased in gastric tumor tissues compared with normal tissues.^64^ In addition, the association analysis between *H. pylori* infection and gastric microbiota has revealed that *H. pylori* abundance can remarkably affect bacterial diversity in gastric cancer tissues.^65^ A significant difference in bacterial microbiota has been observed in non-atrophic gastritis and gastric cancer, and compared with patients with gastritis, *Pseudomonas* is significantly enriched, but the bacterial diversity is reduced in tumor tissues.^66^ Furthermore, *Porphyromonas*, *Neisseria*, *Streptococcus sinensis*, and *TM7* gradually decrease, while *Lactobacillus coleohominis* and *Lachnospiraceae* increase gradually during the development of gastric cancer.^66^ In contrast, another study has shown the relative richen of *Bacilli* and *Streptococcaceae*, whereas the abundance of *Helicobacteraceae* is significantly lower in gastric cancer tissues compared with control samples.^61^ Most researchers have detected species of *Prevotella*, *Veillonella*, *Streptococcus*, *Fusobacterium*, *Neisseria*, and *Haemophilus* in gastric cancer tissues, and have shown the microbiota to be subject-specific and differ among individuals.^67^ It has been reported that CMV, EBV, and HHV6 are detected in gastric tumor tissues, and among them, EBV is the most common one of the genomes that specifically present within the adjacent dysplastic epithelium and gastric carcinoma cells but are absent in surrounding normal cells.^68^, ^69^, ^70^ A recent study has reported that fungal imbalance in tissues is related to gastric cancer, and significant increases in the genera *Alternaria* and *Candida* are observed in gastric cancer tissues compared with non-tumor samples, while other genera, *Thermomyces* and *Saitozyma*, are less abundant in cancer tissues.^71^ So far, the parasitic and archaeal microbiota have not been reported to colonize gastric cancer tissues.

## Intra-tumoral microbiota and colorectal cancer

The composition of fecal microbiota from the gut is not fully concordant with that of the tumor tissue microbiome.^72^ It has been revealed that the bacterial structures of CRC tissues and normal tissues differ significantly, and *Bacteroidetes*, *Firmicutes*, and *Fusobacteria* are increased in cancer tissue compared with normal tissue.^73^, ^74^, ^75^, ^76^ *Lactobacillales*, *Lactococcus*, *Bacteroides*, and *Fusobacterium* are elevated, whereas *Faecalibacterium*, *Pseudomona*s, and *Escherichia-Shigella* are reduced in CRC tissues compared with adjacent normal tissues.^9^^,^^74^^,^^77^ The presence of these bacteria is closely related to histologic grade, poorer overall survival, and resistance to chemotherapy.^74^^,^^78^ *Fusobacterium nucleatum* (*F. nucleatum*), in particular, is enriched in CRC tissue in numerous studies.^9^^,^^78^, ^79^, ^80^ Recent studies have revealed that JC polyomavirus (JCPyV), high-risk HPVs (16, 18, 31, *etc*.), and EBV are widely present in CRC tissues and are considered to contribute to CRC.^81^^,^^82^ The levels of enterovirus 71 (EV71) are higher in CRC tissues than in adjacent non-cancerous tissues and are positively associated with clinical stages (TNM) of CRC patients.^83^ The differences in archaeal composition have been observed between mucosal samples from healthy and diseased tissues from tubular adenoma and adenocarcinoma.^84^ *Methanobacteriales* is a unique group detected in biopsies of CRC, and *Methanobrevibacterium* and *Methanobrevibacter smithii* are the major representative genus and species in CRC tissue.^84^ Until now, it seems that there are still no reports about the colonization of fungi and parasites in CRC tissues.

## Intra-tumoral microbiota and ovarian cancer

Ovarian cancer is the most lethal malignancy of gynecologic cancers, and cannot be diagnosed until the advanced stage of cancer.^16^ Studies have shown that vaginal infections and colonization of the upper genital tract play an important role in the development of ovarian cancer.^85^ Dysbiosis of the vaginal microbiome may promote the development of ovarian cancer by driving local inflammation. Progressive colonization of the vaginal microbiota can promote metastasis in breast cancer. This dysbiosis may provide new ideas for early diagnosis and treatment of ovarian cancer.

Researchers have identified a distinct group of bacterial, viral, parasitic, and fungal signatures of high significance in ovarian cancer.^8^^,^^16^^,^^86^ The microbiome of ovarian tumor tissues is completely different from the surrounding non-tumor tissues, and compared with the control tissues, *Chryseobacterium*, *Enterococcus*, *Francisella*, *Pediococcus*, *Sphingomonas*, *Shewanella*, *Staphylococcus*, and *Treponema* are enriched in ovarian cancer samples.^16^ Additionally, the enrichment of *Brucella*, *Chlamydia*, and *Mycoplasma* has also been highlighted in ovarian cancer tissues.^87^ Viruses have been found in ovarian cancer tissues, mainly human *Papillomavirus* and *Cytomegalovirus*, which may play important roles in ovarian carcinogenesis and progression.^16^^,^^86^^,^^88^^,^^89^ The human *Papillomavirus*, including not only HPV16 and HPV18, but also other HPVs (HPV-2, 4, 5, *etc*.), is detected in ovarian cancer tissues.^16^ Of course, other viruses, including *Poxviridae*, *Retroviridae*, and *Polyomaviridae*, have also been observed in ovarian cancer tissues.^16^ Similar to that seen with viruses, fungal signatures of ovarian tumor tissue are dramatically altered compared with those of control, and *Cladosporium*, *Acremonium*, *Pneumocystis*, *Malassezia*, *Cladophialophora*, and *Microsporidia Pleistophora* are detected only in ovarian cancer tissue samples^16^. The parasitic signature detected in ovarian cancer tissues is far more complex than normal samples, and the presence of *Chlamydia*, *Dipylidium*, *Trichuris*, *Mycoplasma*, and *Leishmania* is found in ovarian cancer tissues.^16^ It has been recently reported that archaea transcript is identified in normal and ovarian cancer tissues,^90^ whereas there is still no evidence on the association between archaea in tissues and ovarian cancer, and the types of archaea are still unclear in ovarian cancer tissues at present.

## Intra-tumoral microbiota and other cancers

In addition to the above-mentioned tumors, it is interesting to note that intra-tumoral microorganisms have also been identified in other cancer types, including melanoma, oral squamous cell carcinoma, liver cancer, esophageal cancer, bone cancer, and glioblastoma multiforme.^8^^,^^12^^,^^91^ In melanoma, *Clostridium* and *Gardnerella vaginalis* exist in the tumor tissue of patients with metastatic melanoma.^8^ In oral squamous cell carcinoma, the intra-tumoral microbiota is dominated by bacterial species that belong to the *Fusobacterium* and *Treponema* genera in tumor tissues.^9^ In liver cancer, HBV and HCV are found to exist in tumor tissues.^91^ Hepatocellular carcinoma tissue possesses a distinct microbiome in comparison to the adjacent tissues, and the tumor-associated microbiota of hepatocellular carcinoma primarily consisted of *Actinobacteria*, *Proteobacteria*, and *Firmicutes*.^92^ In esophageal cancer, detection of *F. nucleatum* in cancer tissues is related to shorter survival, and has a potential role as a prognostic marker.^93^ In bone cancer, *Actinomycetes* and *Lactobacillales* are significantly enriched in tumor tissues.^8^ In glioblastoma multiforme, the *Proteobacteria* phylum is the most abundant bacteria detected in glioblastoma multiforme tissues.^8^ However, very limited evidence has been seen about the association of distinct microbes with these cancer types.

## The summary and characteristics of intra-tumoral microbiota in different cancers

**Figure 1:**
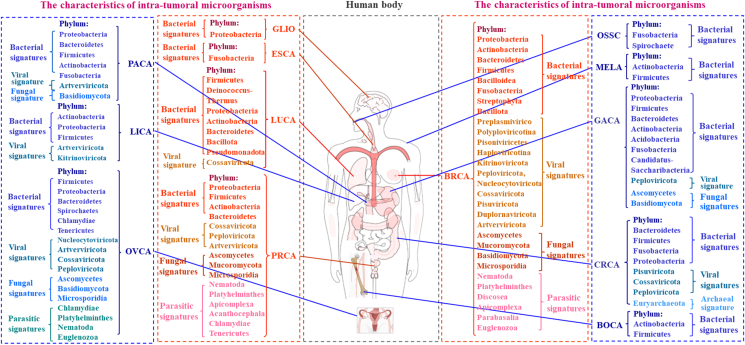
The characteristics of intra-tumoral microbiota in human different cancers. It was summarized based on the current reports of intra-tumoral microbiota in various cancer types, and the intra-tumoral microbiota has significant bacterial, viral, fungal, and parasitic signatures at phylum level. BRCA, breast cancer; PRCA, prostate cancer; OVCA, ovarian cancer; PACA, pancreatic cancer; GACA, gastric cancer; CRCA, colorectal cancer; LUCA, lung cancer; MELA, melanoma; ESCA, esophageal cancer; BOCA, bone cancer; GLIO, glioblastoma multiforme; LICA, liver cancer; OSSA, oral squamous cell carcinoma.

An overview of intra-tumoral microbiota associated with different cancers has been summarized in Table S1, and the phylum, class, and order to which these microbes belong have been determined using NCBI-Taxonomy and confirmed using Bing. To further identify the possible characteristics of intra-tumoral microbiota in different cancers, we analyzed the intra-tumoral microbiota in cancers at phylum, class, and order taxonomic levels. It seems that the intra-tumoral microbiota has distinct characteristics at the phylum level in different cancers. There are significant bacterial, viral, fungal, and parasitic signatures at phylum in breast cancer, prostate cancer, and ovarian cancer; bacterial, viral, and fungal signatures at phylum in pancreatic cancer and gastric cancer; bacterial, viral, and archaeal signatures at phylum in CRC; bacterial and viral signatures at phylum in lung cancer; only bacterial signatures at phylum in melanoma, oral squamous cell carcinoma, esophageal cancer, bone cancer, and glioblastoma multiforme; and only a viral signature at phylum in liver cancer (Table 1 and Fig. 1). The detailed microbiota signatures in different cancers are shown in Table 1 and Figure 1.

**Table 1:** An overview of intra-tumoral microbiota associated with different cancers, with the characteristics of intra-tumoral microbiota at the phylum level.

Cancer types	Subtype	Differential bacterial signatures	Differential viral signatures	Differential fungal signatures	Differential parasitic signatures	Differential archaeal signatures	Reference
Breast cancer	Breast cancer	Phylum:*Proteobacteria*, Phylum:*Actinobacteria*, Phylum:*Bacteroidetes*, Phylum:*Firmicutes*, Phylum: *Bacilloidea*, Phylum:*Fusobacteria*.	Phylum:*Preplasmiviricota*, Phylum:*Polyploviricotina*, Phylum:*Pisoniviricetes*, Phylum:*Haploviricotina*, Phylum:*Kitrinoviricota*, Phylum:*Peploviricota*, Phylum:*Nucleocytoviricota*, Phylum:*Cossaviricota*, Phylum:*Pisuviricota*, Phylum:*Duplornaviricota*, Phylum:*Artverviricota*.	Phylum:*Ascomycetes*, Phylum:*Mucoromycota*, Phylum:*Basidiomycota*, Phylum:*Microsporidia*.	N/A	N/A	20,21,23, 24, 25,27,28
	ER^−^	Phylum:*Actinobacteria*, Phylum:*Streptophyta*, Phylum:*Proteobacteria*, Phylum:*Actinobacteria*.	N/A	N/A	N/A	N/A	
	ER^+^	Phylum:*Actinobacteria*, Phylum:*Proteobacteria*.	N/A	Phylum:*Basidiomycota*, Phylum:*Mucoromycota*, Phylum:*Ascomycetes*.	Phylum:*Nematoda*, Phylum:*Platyhelminthes*.	N/A	
	HER2^+^	Phylum:*Firmicutes*, Phylum:*Bacteroidetes*.	Phylum:*Kitrinoviricota*.	Phylum:*Ascomycetes*.	Phylum:*Discosea*.	N/A	
	TPBC	Phylum:*Proteobacteria*.	Phylum:*Kitrinoviricota*.	Phylum:*Ascomycetes*.	Phylum:*Nematoda*, Phylum:*Platyhelminthes*, Phylum:Apicomplexa, Phylum:*Parabasalia*.	N/A	
	TNBC	Phylum:*Actinobacteria*, Phylum:*Firmicutes*, Phylum:*Proteobacteria*, Phylum:*Fusobacteria*, Phylum:*Streptophyta*, Phylum:*Bacillota*.	Phylum:*Peploviricota*, Phylum:*Artverviricota*, Phylum:*Nucleocytoviricota*, Phylum:*Cossaviricota*.	Phylum:*Ascomycetes*, Phylum:*Basidiomycota*, Phylum:*Mucoromycota*, Phylum:*Microsporidia*.	Phylum:*Platyhelminthes*, Phylum:*Nematoda*, Phylum:*Euglenozoa*.	N/A	
Lung cancer		Phylum:*Firmicutes*, Phylum:*Deinococcus*-*Thermus*, Phylum:*Proteobacteria*, Phylum:*Actinobacteria*, Phylum:*Bacteroidetes*, Phylum:*Bacillota*, Phylum:*Pseudomonadota*.	Phylum:*Cossaviricota*.	N/A	N/A	N/A	30,36
Prostate cancer		Phylum:*Proteobacteria*, Phylum:*Firmicutes*, Phylum:*Actinobacteria*, Phylum:*Bacteroidetes*.	Phylum:*Cossaviricota*, Phylum:*Peploviricota*, Phylum:*Artverviricota*.	Phylum:*Ascomycetes*, Phylum:*Mucoromycota*, Phylum:*Microsporidia*.	Phylum:*Nematoda*, Phylum:*Platyhelminthes*, Phylum:*Apicomplexa*, Phylum:*Acanthocephala*, Phylum:*Chlamydiae*, Phylum:*Tenericutes*.	N/A	24,37,38,40,47,48
Pancreatic cancer		Phylum:*Proteobacteria*, Phylum:*Bacteroidetes*, Phylum:*Firmicutes*, Phylum:*Actinobacteria*, Phylum:*Fusobacteria*.	Phylum:*Artverviricota*.	Phylum:*Basidiomycota*.	N/A	N/A	17,52, 53, 54,56
Gastric cancer		Phylum:*Proteobacteria*, Phylum:*Firmicutes*, Phylum:*Bacteroidetes*, Phylum:*Actinobacteria*, Phylum:*Acidobacteria*, Phylum:*Fusobacteria*, Phylum:*Candidatus-Saccharibacteria* (*TM7*).	Phylum:*Peploviricota*.	Phylum:*Ascomycetes*, Phylum:*Basidiomycota*.	N/A	N/A	57, 58, 59, 60, 61, 62, 63, 64, 65, 66, 67, 68, 69, 70
Colorectal cancer		Phylum:*Bacteroidetes*, Phylum:*Firmicutes*, Phylum:*Fusobacteria*, Phylum:*Proteobacteria*.	Phylum:*Pisuviricota*, Phylum:*Cossaviricota*, Phylum:*Peploviricota*.	N/A	N/A	Phylum:*Euryarchaeota*.	71, 72, 73, 74, 75, 76, 77,80, 81, 82, 83
Ovarian cancer		Phylum:*Firmicutes*, Phylum:*Proteobacteria*, Phylum:*Bacteroidetes*, Phylum:*Spirochaetes*, Phylum:*Chlamydiae*, Phylum:*Tenericutes*.	Phylum:*Nucleocytoviricota*, Phylum:*Artverviricota*, Phylum:*Cossaviricota*, Phylum:*Peploviricota*.	Phylum:*Ascomycetes*, Phylum:*Basidiomycota*, Phylum:*Microsporidia*.	Phylum:*Chlamydiae*, Phylum:*Platyhelminthes*, Phylum:*Nematoda*, Phylum:*Euglenozoa*.	N/A	16,84,86,87
Melanoma		Phylum:*Firmicutes*, Phylum:*Actinobacteria*.	N/A	N/A	N/A	N/A	8
Oral squamous cell carcinoma		Phylum:*Fusobacteria*, Phylum:*Spirochaete*	N/A	N/A	N/A	N/A	9
Liver cancer		Phylum:*Actinobacteria*, Phylum:*Proteobacteria,* Phylum:*Firmicutes*	Phylum:*Artverviricota*, Phylum:*Kitrinoviricota*.	N/A	N/A	N/A	89,90
Esophageal cancer		Phylum:*Fusobacteria*.	N/A	N/A	N/A	N/A	91
Bone cancer		Phylum:*Actinobacteria*, Phylum:*Firmicutes*.	N/A	N/A	N/A	N/A	8

### The mechanisms of intra-tumoral microbiota in cancers

It has been clarified that there is an inseparable connection between intra-tumoral microbiota and the occurrence and development of cancers, and several mechanisms have been revealed as potential modes of action.^94^ The tumor microenvironment and cancer pathogenesis are intricately linked. Tumor-associated microbes can induce genomic instability, impact epigenetic modifications, modulate the host’s immune response, and interact with the tumor microenvironment to activate carcinogenic signaling pathways such as invasion and metastasis, thereby promoting cancer occurrence and progression.^95^ Research has demonstrated that tumor-associated microbes can enhance genetic instability in cancer cells through mechanisms such as DNA damage or interference with DNA repair processes, ultimately influencing tumor advancement.^95^ In addition to directly regulating cancer cells, the presence of microbes within the tumor can also modify the tumor microenvironment and modulate the host’s immune response, thereby impacting tumor metastasis.^96^ Certain bacteria within the tumor influence the growth and activity of other microbes through their metabolic products, thus governing the tumor microenvironment and subsequently influencing the cancer process.^97^ Moreover, tumor-associated microbes can also impact cancer progression by participating in the regulation of metabolic processes in cancer cells and mediating changes in the secretion of cancer-related exosomes, which in turn regulate cancer-associated signaling pathways.^98^^,^^99^

**Figure 2:**
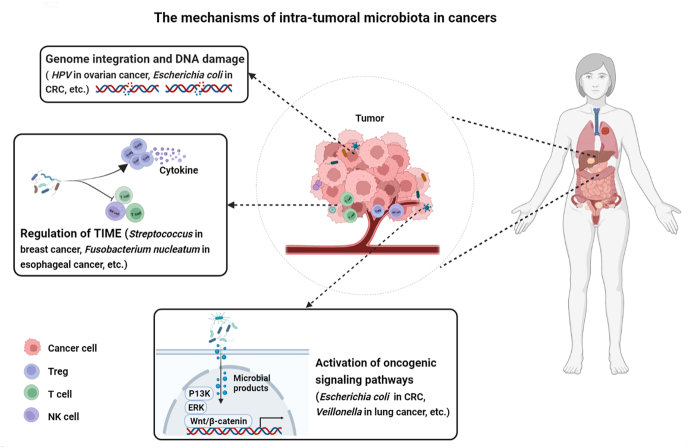
The mechanisms of intra-tumoral microbiota in human cancers. The intra-microbial-mediated mechanisms in cancer are three major types: promotion of genome integration and DNA damage, activation of oncogenic signaling pathways, and regulation of tumor immune micro-environment (TIME). Created with BioRender.com.

Currently, the major mechanisms have been proposed to elucidate the impact of intra-tumoral microbiota on the initiation and progression of cancers: facilitation of genome integration and DNA damage, activation of oncogenic signaling pathways, and modulation of tumor immune microenvironment (Fig. 2).

## The intra-tumoral microbiota involved tumors by genome integration and DNA damage

Intra-tumoral microbiota can directly affect the cancer process through genome integration. In ovarian cancer tissues, viral sequences can be widely integrated into the genome of host tumor cells, for example, HPV gene E1/E2/E5 regions can be integrated at various intronic regions of host genes as well as upstream promoter regions, which may potentially act roles on development and progression of ovarian cancer.^16^ Another virus integration site of interest is the coding sequences of the HHVI UL42 gene that can be inserted into the intronic region of the neogenin (NEO1) gene.^16^ Low expression of NEO1 has been observed in various cancers, and the altered expression of NEO1 resulting in loss of pro-apoptotic activity plays important roles in tumorigenesis.^100^^,^^101^ Intra-tumoral microbiota possesses the ability to produce compounds that cause DNA damage and genetic instability.^94^ For example, the production of colicins in some *Escherichia coli* (*E. coli*) and other *Enterobacteriaceae* can cause double-stranded DNA breaks, and thereby promote the tumorigenesis of CRC.^102^

## The intra-tumoral microbiota involved tumors by activating oncogenic signaling pathways

Aside from direct DNA damage, intra-tumoral microbiota products can also participate in host oncogenic pathways, for example, *E. coli* products can up-regulate Wnt/β-catenin signaling pathway leading to carcinogenesis.^102^ In gastric cancer and CRC, local commensal microbiota can activate β-catenin expression, then facilitate transcription of oncogenes like cyclin D-1 and c-Myc to advance carcinogenesis and progression of cancer.^103^, ^104^, ^105^ In lung cancer, intra-tumoral microbiome dysbiosis, such as *Veillonella* and *Streptococcus*, may up-regulate the extracellular signal-regulated kinase (ERK) and phosphatidylinositol 3-kinase (PI3K) pathway, and thereby promote carcinogenesis.^94^ In addition, intracellular microbiota plays a key role in tumor metastasis by regulating the cytoskeleton and cell viability under mechanical stress in breast cancer.^10^

## The intra-tumoral microbiota involved tumors by regulating the tumor immune microenvironment

The microbiota in tumor tissue constitutes an important part of the tumor microenvironment, affecting tumor occurrence and progression in a more local area. In tumor tissues, the balance between microbiome and host immune system is disrupted, and the changes of intra-tumoral microbiome cause dysbiosis of the normal microenvironment to accelerate the progression of cancers.^94^ Relevant studies have shown that bacteria in breast cancer tissues affect tumorigenesis by regulating the immune microenvironment, and the variation of microbial diversity in breast tumors disrupts homeostatic microbiome–immune interactions, resulting in immune dysregulation and carcinogenesis.^21^^,^^106^ Aside from affecting host immune responses directly, the microbiome of breast tissue may also produce metabolites that influence cancer and immune cells. In breast cancer, the species of *Streptococcus* genus, which is present at much lower abundance in tumor tissue, can synthesize cadaverine (a lysine derivative) that represses epithelial-to-mesenchymal transition and tumor invasion,^20^^,^^107^ and microbiota-derived bile acids can be accumulated in breast tumor tissues, which is correlated with decreased proliferation of tumor cells.^108^ In esophageal cancer, *F. nucleatum* in tumor tissues promotes tumor infiltration of regulatory T lymphocytes in a chemokine-dependent manner, and thereby facilitates aggressive tumor behavior.^93^ Intra-tumoral bacteria can alter the activation of regional immune cells and recruitment, influencing the development and progression of cancer. The bacteria *F. nucleatum* existing in the tumor microenvironment can inhibit the activity of immune cells, such as various T cells and natural killer cells.^109^ In CRC, *F. nucleatum* can produce an immunosuppressive tumor microenvironment, reflected in the inverse correlation between its abundance and CD3^+^ T cell density in tumor tissue of CRC.^110^ In some cases, intra-tumoral bacteria may directly influence the immune activation of the tumor microenvironment, such as the *F. nucleatum* in melanoma that drives human leukocyte antigen presentation of bacterial peptides.^111^ In addition, recent reports have proved that the microbiome in breast tissue produces metabolites that are different from the gut microbiome,^112^ suggesting different functions and mechanisms of these different metabolites in breast cancer tissues.

### The values of intra-tumoral microbiota in cancer diagnosis and prognosis

The important characteristics of malignant tumors are poor prognosis and clinical outcomes, which are largely due to the lack of early diagnostic markers and powerful therapies.^113^, ^114^, ^115^, ^116^, ^117^, ^118^ Therefore, there is an urgent requirement to find early diagnostic markers with high accuracy and new treatment strategies to improve the prognosis of cancer patients. The strong connections between intra-tumoral microbiota and various tumors suggest that intra-tumoral microbiota have the potential to serve as novel diagnostic or prognostic markers. Recently, many studies have begun to reveal the diagnostic value and therapeutic potential of intra-tumoral microbiota. More and more evidences demonstrate that the intra-tumoral microbiota can be used as a promising diagnostic and prognostic biomarker to provide guidelines for the prevention and treatment of different tumors. Testing the composition of the microbiome for cancer diagnosis has been raised in a few cancer types.

## The intra-tumoral microbiota and cancer (early) diagnosis

As a component of tumors, intra-tumoral microbiota have been shown to have the potential to be novel diagnostic markers. The unique microbial signatures have been found in tissue and blood within and between most major cancer types, and the tumor tissues are successfully distinguished from normal tissue by microbial composition, revealing the potential diagnostic value of detecting intra-tumoral microbiome.^91^ For example, *F. nucleatum* has been shown as a potential diagnostic biomarker in cervical carcinoma.^119^

## The intra-tumoral microbiota and prognosis of cancer patients

As a component of tumors, intra-tumoral microbiota have also been found to possess the potential as prognostic markers, and the prognostic value of intra-tumoral microbiota in cancers has received extensive attention from researchers. In cervical carcinoma, *F. nucleatum* could be a potential prognostic biomarker.^109^ In lung cancer, higher bacterial microbiome diversity and composition in normal lung tissues are associated with the survival of lung cancer patients. The greater bacterial abundance of families *Lachnospiraceae*, *Ruminococcaceae*, and *Bacteroidaceae*, and genera *Bacteroides*, *Ruminococcus*, *Roseburia*, and *Faecalibacterium* in normal lung tissues is associated with reduced disease-free and recurrence-free survival of lung cancer patients, while the greater abundance of *Sphingomonadaceae* and *Koribacteraceae* is associated with increased disease-free survival of lung cancer patients.^33^ Higher abundance of *Koribacteraceae* is associated with increased recurrence-free survival and disease-free survival of lung cancer patients, while higher abundance of *Bacteroidaceae*, *Ruminococcaceae*, and *Lachnospiraceae* is related to reduced survival of lung cancer patients.^33^ In nasopharyngeal carcinoma, patients with a higher intra-tumoral bacterial load had inferior rates of disease-free survival, distant metastasis-free survival, and overall survival than those with a low bacterial load.^13^ In esophageal squamous cell carcinoma, high levels of intra-tumoral *F. nucleatum* indicated poor prognosis in patients.^120^ Moreover, the higher number of *F. nucleatum* in CRC tissues is associated with poor survival of metastatic CRC patients.^78^^,^^121^ High levels of *F. nucleatum* were also associated with poorer overall survival and disease-free survival in cervical carcinoma patients.^119^ High levels of *Akkermansia* and *Methylobacterium* in hepatocellular carcinoma tissues were associated with a favorable prognosis, suggesting that *Akkermansia* and *Methylobacterium* may be significant prognostic markers for hepatocellular carcinoma.^92^ The presence of *Methylobacterium* within gastric cancer tumors was associated with poor prognosis of cancer patients.^122^ In addition, some intra-tumoral microbes, such as *Fusobacterium*, *Pseudoxanthomonas*, *Streptomyces*, *Saccharopolyspora*, and *Bacillus clausii*, are associated with the prognosis of pancreatic cancer,^53^^,^^54^ indicating that these microbes may be prognostic biomarkers for pancreatic cancer patients.

Until now, the microorganisms thought to be promising diagnostic and prognostic biomarkers have been summarized in Table 2.

**Table 2:** An overview of intra-tumoral microbiota reported to be promising diagnostic and prognostic biomarkers.

Cancer types	Diagnostic biomarkers					Prognostic biomarkers				
	Bacterial	Viral	Fungal	Parasitic	Archaeal	Bacterial	Viral	Fungal	Parasitic	Archaeal
Cervical carcinoma	*Fusobacterium nucleatum*^119^	/	/	/	/	*Fusobacterium nucleatum*^119^	/	/	/	/
Lung cancer	/	/	/	/	/	*Streptococcus viridans*, *Granulicatella adiacens*, *Koribacteraceae*, *Bacteroidaceae*, *Lachnospiraceae*, *Ruminococcaceae*, *Bacteroides*, *Faecalibacterium*, *Ruminococcus*, *Sphingomonadaceae, Roseburia*^33^	/	/	/	/
Hepatocellular carcinoma	/	/	/	/	/	*Akkermansia*, *Methylobacterium*^92^	HBV	/	/	/
Pancreatic cancer	/	/	/	/	/	*Fusobacterium*, *Pseudoxanthomonas*, *Streptomyces*, *Saccharopolyspora*, *Bacillus clausii*^53^^,^^54^	/	/	/	/
Esophageal squamous cell carcinoma	/	/	/	/	/	*Fusobacterium nucleatum*^120^	/	/	/	/
Colorectal cancer	/	/	/	/	/	*Fusobacterium nucleatum*^77^^,^^121^	/	/	/	/
Gastric cancer	/	/	/	/	/	*Methylobacterium*^122^	/	/	/	/

### The values of intra-tumoral microbiota in cancer therapy and precision medicine

In the past few decades, although the continuous progress of cancer treatment methods has greatly improved the treatment effectiveness of cancer patients, limitations such as drug resistance and toxicity still exist. More and more evidences suggest a potential link between tumor microbiota and cancer treatment efficacy.^11^^,^^95^^,^^123^ The microbiota within tumors can significantly affect the toxicity and regulate the efficacy of cancer therapies, including chemotherapy, immunotherapy, and radiation therapy.^11^^,^^124^ Increasing evidence suggests that tumor microbiota is a novel and important adjunct to conventional anti-cancer therapy, providing more strategies for cancer treatment.

## The intra-tumoral microbiota and cancer immunotherapeutics

Although immunotherapy plays a crucial role in improving the prognosis of tumor patients, a large percentage of tumor patients still fail to benefit from immunotherapy. Several studies have shown that the presence of intra-tumor microbiota can influence the efficacy of cancer immunotherapy by participating in immune regulation.^125^, ^126^, ^127^ Currently, immune checkpoint inhibitor therapy is a core component of cancer treatment, demonstrating unprecedented efficacy in some cancer patients. Monoclonal antibodies against programmed death protein 1 (PD-1)/programmed death ligand 1 (PD-L1) and cytotoxic T-lymphocyte-associated protein 4 (CTLA-4) are widely used in clinical practice. Intra-tumoral microbiota can generally suppress the immune response and promote cancer progression, whereas some studies have shown that intra-tumoral microbiota clearly enhance the efficacy of immunotherapy.^126^^,^^128^ The metabolite trimethylamine oxide (TMAO) of *Clostridiales* promotes M1 macrophage infiltration and CD8 T cell activation, enhances CD8 T-mediated anti-tumor immunity, and thereby improves anti-PD-1 therapeutic response in TNBC.^125^ The *Lactobacillus reuteri* in melanoma can release dietary tryptophan catabolite I3A, which promotes the production of interferon-γ CD8 T cells, and thereby enhances the immune checkpoint inhibitor treatment of tumor tissue.^126^ In a mouse model of colon adenocarcinoma, *Bifidobacterium* promotes local anti-CD47 immunotherapy on tumor tissue through the capacity to accumulate in the tumor microenvironment.^128^ A recent report has also shown that commensal *Bifidobacterium* confer protection to melanoma patients by promoting anti-PD-L1 therapy.^129^ These pieces of evidence suggest that the microbiota within tumors can play positive roles in cancer patients by enhancing the effect of immunotherapy, providing new hope for improving the efficacy of immunotherapy.

## The intra-tumoral microbiota and cancer chemotherapeutics and radiotherapeutics

Although chemotherapy and radiation therapy are the main means of cancer treatment, they have significant limitations, including a lack of specificity and accuracy, inability to accurately distinguish between tumors and healthy tissues, and especially resistance. In most cases, the tumor microbiota is often the cause of chemotherapy drug resistance. The intra-tumoral bacteria *Gammaproteobacteria* of pancreatic cancer may play a crucial role in mediating the drug resistance of gemcitabine therapy by producing cytidine deaminase, leading to gemcitabine degradation.^52^ Increased levels of oral pathogens, *Aggregatibacter actinomycetemcomitans* and *P. gingivalis*, have been observed in pancreatic cancer patients, which promotes the expression of cytidine deaminase to impact the occurrence of chemoresistance.^130^ In CRC patients, *F. nucleatum* directly promotes CRC chemoresistance to oxaliplatin and 5-fluorouracil through autophagy or Toll-like receptor 4 (TLR4)/myeloid differentiation primary-response protein 88 (MYD88)-dependent mechanism.^78^^,^^121^ Similarly, the relative abundance of *F*. *nucleatum* is higher in pancreatic cancer tissues than in non-cancer subjects, which can also cause drug resistance of pancreatic cancer through the MYD88 pathway.^131^, ^132^ *Lactobacillus iners*, found in cervical cancer, can induce chemotherapy and radiation resistance through reprogramming metabolic signaling pathways.^124^ However, only a small portion of the tumor microbiota has been proven to alleviate the side effects of radiotherapy. The binding of *Candida albicans* to dectin-1 receptor up-regulates tumor-promoting macrophages and down-regulates anti-tumor T cells, and thereby inhibits anti-tumor immune response after radiotherapy in mouse breast cancer.^133^ Oral microbiota dysbiosis contributes to exacerbating mucositis severity in nasopharyngeal carcinoma patients undergoing radiotherapy, and *Streptococcus mitis* is significantly increased in nasopharyngeal carcinoma patients after irradiation.^134^ With the research on the role of tumor microbiota in chemoradiotherapy resistance, new auxiliary strategies can be provided for anti-tumor treatment by targeting microbiota.

These studies indicate that intra-tumoral microbiota influence tumor development and treatment. In the context of cancer treatment resistance, the microbiome has been implicated in influencing the efficacy of cancer therapies, including chemotherapy and immunotherapy. Preclinical and clinical studies have highlighted the critical role of the microbiome in determining the effectiveness of cancer treatments, emphasizing the need to consider the microbiome in therapeutic strategies.^135^

The tumor microenvironment has been increasingly recognized as a critical factor in the development of resistance to chemotherapy in solid tumors.^136^ Specific bacteria, such as *F. nucleatum*, have been found to promote breast cancer cell stemness and chemoresistance through interactions with immune signaling pathways.^137^ The diversity of the tumor microbiota is also influenced by chemotherapy and immunotherapy, with genetically engineered bacteria showing promise in tumor treatment.^138^ Microbiota have been identified as crucial players in the development of resistance to cancer chemoradiotherapy, underscoring the need for a deeper understanding of their role in treatment outcomes.^139^ Chemotherapy resistance can also be influenced by disruptions in the abundance and metabolism of intra-tumoral microbiota, which can directly interfere with the anti-tumor outcomes of various cancer therapies.^140^ Strategies such as photodynamic eradication of intra-tumoral microbiota have been proposed to overcome resistance in pancreatic cancer, pointing towards innovative approaches to address this challenge.^141^

Furthermore, the use of prebiotics, probiotics, or fecal transplantation has been suggested as a means to modulate intestinal and vaginal dysbiosis in endometrial cancer, potentially improving treatment outcomes and quality of life.^142^ The correlation between immune characteristics, methylation patterns, tumor heterogeneity, and tumor stemness underscores the multifactorial nature of chemotherapy resistance in breast cancer and the need for comprehensive approaches to address it.^143^ Activatable polymer nanoenzymes have been proposed for immunometabolic cancer therapy, targeting metabolic pathways that influence tumor progression and treatment responses.^144^

In conclusion, the tumor microbiota plays a crucial role in mediating chemotherapy resistance through complex interactions with the tumor microenvironment, immune system, and treatment modalities. Understanding and targeting these microbial populations hold promise for enhancing the effectiveness of cancer therapies and overcoming resistance mechanisms. Innovative approaches that leverage microbiota modulation, genetic engineering, and immunometabolic interventions may pave the way for more personalized and effective cancer treatments in the future.

### The applications of intra-tumoral microbiota in cancer precision medicine

Clinical studies for altering microbiota to inhibit oncogenesis have shown promising results. The modulation of intra-tumoral microbiota may be a new and important adjunct for current anti-cancer therapies. With the continuous advancement of nanomaterials and bioengineering technology, new methods of cancer treatment, including genetic engineering, have become possible.^145^ Recently, nanotechnologies have been used to act on tumor-associated bacteria and their metabolites at a vascular-accessible tumor site to enable or facilitate cancer therapy.^145^, ^146^ Several intra-tumoral microbiota, including *Bifidobacterium*, *Salmonella*, *Clostridium*, *Listeria*, and *Escherichia coli*, that colonize and accumulate in tumors, can be successfully used as an *in vivo* delivery vehicle for cancer treatment.^147^ *Listeria monocytogenes* is a gram-positive facultative anaerobic bacterium, and its attenuated strain has been safely used as a therapeutic bacterial carrier for the delivery of cancer vaccines, which is widely used in various cancer treatments, including cervical cancer, pancreatic cancer, prostate cancer, breast cancer, lung cancer, and mesothelioma.^148^ *Clostridium* is a strictly anaerobic bacterium that can colonize tumor tissues and has been used for tumor treatment in recent years.^8^^,^^149^ A non-pathogenic *E. coli* strain has been designed with the capability of synthesizing CD47 nanobody antagonists in the tumor microenvironment, increasing the activation of tumor-infiltrating T cells, stimulating rapid tumor regression, and preventing metastasis.^150^ The *E. coli* strain is bioengineered to effectively increase the concentration of l-arginine and enhance T cell infiltration in the tumor microenvironment, thereby enhancing the efficacy of immunotherapy.^151^ The *Salmonella* can accumulate around tumors, penetrate the cell barrier, and replicate within tumors, where they deliver anti-cancer agents or pro-apoptotic genes to achieve cancer therapeutic effects.^152^, ^153^ Although cancer therapy based on intra-tumoral microbiota has good application prospects, the progress of live bacterial therapy in clinical applications is slow. To date, only *Bacillus* Calmette-Guérin (BCG) and attenuating *Mycobacterium bovis* have been used clinically as bacterial-based cancer therapies in bladder cancer.^154^ In general, research on the intra-tumoral microbiome has raised widespread expectations to improve the lives of cancer patients. In this case, precision medicine targeting the microbiota will become one of the next frontier fields in cancer treatment, providing tailored treatment opportunities for individual cancer patients. However, there is currently no application of microbiome in precision therapy research, which requires further studies in the future.

The major precise roles of intra-tumoral microbiota in different therapies for tumors have been summarized in Table 3.

**Table 3:** An overview of the major precise roles of microbiota in different therapies of tumors.

Therapy types	Cancer types	Microbial types	Function
Immuno-therapy	Breast cancer	*Clostridiales*	Production of metabolite trimethylamine enhances anti-programmed death protein 1 (PD-1) immunotherapy
	Melanoma	*Lactobacillus reuteri*	Enhance the therapeutic efficacy of immune checkpoint inhibitors through the released dietary tryptophan catabolite I3A
		*Bifidobacterium*	Improve basic tumor control and anti-PD-l efficacy
	Colon adenocarcinoma	*Bifidobacterium*	Promote local anti-CD47 immunotherapy
Chemo-therapy	Colorectal cancer	*Fusobacterium nucleatum*	Induce drug resistance to oxaliplatin and 5-fluorouracil in colorectal cancer
	Cervical cancer	*Lactobacillus iners*	Induce chemotherapy and radiation resistance through reprogramming metabolic signaling pathways
	Pancreatic cancer	*Fusobacterium nucleatum*	Cause the drug resistance through the myeloid differentiation primary-response protein 88 (MYD88) pathway
		*Aggregatibacter actinomycetemcomitans*, *Porphyromonas gingivalis*	Impact the occurrence of chemoresistance
		*Gamma-Proteobacteria*	Lead to gemcitabine degradation and the development of resistance
Radio-therapy	Nasopharyngeal carcinoma	*Streptococcus mitis*	Contribute to exacerbating the severity of mucositis in patients undergoing radiotherapy
	Breast cancer	*Candida albicans*	Inhibit anti-tumor immune response after radiotherapy

### Discussion and prospect

Most major types of human cancer contain tumor microbiota. The intra-tumoral microbiota observed in different cancer types and sample types is usually different. This phenomenon indicates that the occurrence and progression of tumors are related to the imbalance of tumor microbial diversity and stability. It is becoming increasingly apparent that both relative abundance and community composition of bacteria are contributory factors to cancers. Studies of different tumor types have shown that the intra-tumoral microbiota of the same tumor type tends to be more similar to each other than that of other tumor types. The distribution of order-level phylotypes shows significant changes in bacterial compositions of different tumor types. These results suggest that different intra-tumoral microbiota may play different roles or biases in different tumor types or different organizational system tumors.

Although our understanding of the microbiome in cancer is growing, there is currently little direct evidence that intra-tumoral microbiota is a crucial determinant of cancer pathogenesis. Whether the presence of microbiota in tumor samples is a causative factor or a consequence of a dysfunctional tumor environment remains an open question in most cases. Not only that, most researches are focused on the roles of microbiome, and few studies on the functional mechanism have been carried out at present.

Intra-tumoral microbiota is an important component of the tumor microenvironment, and its key role in regulating tumor occurrence and progression and influencing the prognosis of tumor patients in the tumor microenvironment has attracted more and more attention. However, the types of microorganisms within the tumor and the specific host cell types they interact with in different tumors have not been fully revealed. In addition, it is largely unknown whether the spatial distribution of tumor microbiota and specific host microbial cell interactions affect the different functions of the tumor microenvironment. The mechanism by which intra-tumoral microbiota participate in tumor immunotherapy response is still unclear, which limits the clinical application of microbial-related treatment strategies in tumors. Therefore, extensive validation through animal models and clinical trials is still needed, and we believe that in the future, successful treatment of tumors can be achieved using microbiota, targeted microbiota, or in combination with immunotherapy.

In summary, this article mainly summarizes the characteristics and roles of intra-tumoral microbiota in different tumor types, highlights the key mechanisms by which tumor microbiota affects tumor occurrence and development, and discusses the diagnostic, prognostic, and therapeutic potential of intra-tumoral microbiota in tumors. We hope our review can provide some ideas for further innovation and clinical applications in the field of cancer microorganisms.

## CRediT authorship contribution statement

**Tingting Zhao:** Data curation, Investigation, Writing – original draft, Writing – review & editing. **Na Sun:** Data curation, Investigation, Writing – review & editing. **Jun Ding:** Data curation, Investigation. **Zaihui Peng:** Data curation. **Fei Han:** Funding acquisition, Writing – original draft, Writing – review & editing. **Xiaowei Qi:** Conceptualization, Funding acquisition, Writing – review & editing.

## Conflict of interests

The authors declared no conflict of interests.

## References

[1] Ursell L.K., Metcalf J.L., Parfrey L.W., Knight R. (2012). Defining the human microbiome. Nutr Rev.

[2] Cani P.D. (2018). Human gut microbiome: hopes, threats and promises. Gut.

[3] Helmink B.A., Wadud Khan M.A., Hermann A., Gopalakrishnan V., Wargo J.A. (2019). The microbiome, cancer, and cancer therapy. Nat Med.

[4] Li J., Zhao F., Wang Y. (2017). Gut microbiota dysbiosis contributes to the development of hypertension. Microbiome.

[5] de Martel C., Georges D., Bray F., Ferlay J., Clifford G.M. (2020). Global burden of cancer attributable to infections in 2018: a worldwide incidence analysis. Lancet Global Health.

[6] Picardo S.L., Coburn B., Hansen A.R. (2019). The microbiome and cancer for clinicians. Crit Rev Oncol Hematol.

[7] Cullin N., Azevedo Antunes C., Straussman R., Stein-Thoeringer C.K., Elinav E. (2021). Microbiome and cancer. Cancer Cell.

[8] Nejman D., Livyatan I., Fuks G. (2020). The human tumor microbiome is composed of tumor type-specific intracellular bacteria. Science.

[9] Galeano Niño JL., Wu H., LaCourse K.D. (2022). Effect of the intratumoral microbiota on spatial and cellular heterogeneity in cancer. Nature.

[10] Fu A., Yao B., Dong T. (2022). Tumor-resident intracellular microbiota promotes metastatic colonization in breast cancer. Cell.

[11] Xue C., Chu Q., Zheng Q. (2023). Current understanding of the intratumoral microbiome in various tumors. Cell Rep Med.

[12] Liu Y., Lin Z., Lin Y. (2018). *Streptococcus* and *Prevotella* are associated with the prognosis of oesophageal squamous cell carcinoma. J Med Microbiol.

[13] Qiao H., Tan X.R., Li H. (2022). Association of intratumoral microbiota with prognosis in patients with nasopharyngeal carcinoma from 2 hospitals in China. JAMA Oncol.

[14] Ferreira R.M., Pereira-Marques J., Pinto-Ribeiro I. (2018). Gastric microbial community profiling reveals a dysbiotic cancer-associated microbiota. Gut.

[15] Urbaniak C., Gloor G.B., Brackstone M., Scott L., Tangney M., Reid G. (2016). The microbiota of breast tissue and its association with breast cancer. Appl Environ Microbiol.

[16] Banerjee S., Tian T., Wei Z. (2017). The ovarian cancer oncobiome. Oncotarget.

[17] Pushalkar S., Hundeyin M., Daley D. (2018). The pancreatic cancer microbiome promotes oncogenesis by induction of innate and adaptive immune suppression. Cancer Discov.

[18] Sfanos K.S., Sauvageot J., Fedor H.L., Dick J.D., De Marzo A.M., Isaacs W.B. (2008). A molecular analysis of prokaryotic and viral DNA sequences in prostate tissue from patients with prostate cancer indicates the presence of multiple and diverse microorganisms. Prostate.

[19] Avilés-Jiménez F., Guitron A., Segura-López F. (2016). Microbiota studies in the bile duct strongly suggest a role for *Helicobacter pylori* in extrahepatic cholangiocarcinoma. Clin Microbiol Infect.

[20] Mikó E., Kovács T., Sebő É. (2019). Microbiome-microbial metabolome-cancer cell interactions in breast cancer - familiar, but unexplored. Cells.

[21] Xuan C., Shamonki J.M., Chung A. (2014). Microbial dysbiosis is associated with human breast cancer. PLoS One.

[22] Hieken T.J., Chen J., Hoskin T.L. (2016). The microbiome of aseptically collected human breast tissue in benign and malignant disease. Sci Rep.

[23] Meng S., Chen B., Yang J. (2018). Study of microbiomes in aseptically collected samples of human breast tissue using needle biopsy and the potential role of *in situ* tissue microbiomes for promoting malignancy. Front Oncol.

[24] Banerjee S., Tian T., Wei Z. (2018). Distinct microbial signatures associated with different breast cancer types. Front Microbiol.

[25] Banerjee S., Wei Z., Tan F. (2015). Distinct microbiological signatures associated with triple negative breast cancer. Sci Rep.

[26] Wang Y., Qu D., Zhang Y. (2023). Intra-tumoral microbial community profiling and associated metabolites alterations of TNBC. Front Oncol.

[27] Urbaniak C., Cummins J., Brackstone M. (2014). Microbiota of human breast tissue. Appl Environ Microbiol.

[28] Alpuim Costa D., Nobre J.G., Batista M.V. (2021). Human microbiota and breast cancer - is there any relevant link?-A literature review and new horizons toward personalised medicine. Front Microbiol.

[29] Shively C.A., Register T.C., Appt S.E. (2018). Consumption of mediterranean versus western diet leads to distinct mammary gland microbiome populations. Cell Rep.

[30] Yu G., Gail M.H., Consonni D. (2016). Characterizing human lung tissue microbiota and its relationship to epidemiological and clinical features. Genome Biol.

[31] Greathouse K.L., White J.R., Vargas A.J. (2018). Interaction between the microbiome and TP53 in human lung cancer. Genome Biol.

[32] Liu H.X., Tao L.L., Zhang J. (2018). Difference of lower airway microbiome in bilateral protected specimen brush between lung cancer patients with unilateral lobar masses and control subjects. Int J Cancer.

[33] Peters B.A., Hayes R.B., Goparaju C., Reid C., Pass H.I., Ahn J. (2019). The microbiome in lung cancer tissue and recurrence-free survival. Cancer Epidemiol Biomark Prev.

[34] Mao Q., Ma W., Wang Z. (2020). Differential flora in the microenvironment of lung tumor and paired adjacent normal tissues. Carcinogenesis.

[35] Dumont-Leblond N., Veillette M., Racine C., Joubert P., Duchaine C. (2021). Non-small cell lung cancer microbiota characterization: prevalence of enteric and potentially pathogenic bacteria in cancer tissues. PLoS One.

[36] Cai H.Z., Zhang H., Yang J., Zeng J., Wang H. (2021). Preliminary assessment of viral metagenome from cancer tissue and blood from patients with lung adenocarcinoma. J Med Virol.

[37] Banerjee S., Alwine J.C., Wei Z. (2019). Microbiome signatures in prostate cancer. Carcinogenesis.

[38] Ohadian Moghadam S., Momeni S.A. (2021). Human microbiome and prostate cancer development: current insights into the prevention and treatment. Front Med.

[39] Porter C.M., Shrestha E., Peiffer L.B., Sfanos K.S. (2018). The microbiome in prostate inflammation and prostate cancer. Prostate Cancer Prostatic Dis.

[40] Wheeler K.M., Liss M.A. (2019). The microbiome and prostate cancer risk. Curr Urol Rep.

[41] Katongole P., Sande O.J., Joloba M., Reynolds S.J., Niyonzima N. (2020). The human microbiome and its link in prostate cancer risk and pathogenesis. Infect Agents Cancer.

[42] Yow M.A., Tabrizi S.N., Severi G. (2017). Characterisation of microbial communities within aggressive prostate cancer tissues. Infect Agents Cancer.

[43] Cavarretta I., Ferrarese R., Cazzaniga W. (2017). The microbiome of the prostate tumor microenvironment. Eur Urol.

[44] Feng Y., Ramnarine V.R., Bell R. (2019). Metagenomic and metatranscriptomic analysis of human prostate microbiota from patients with prostate cancer. BMC Genom.

[45] Al-Marhoon M.S., Ouhtit A., Al-Abri A.O. (2015). Molecular evidence of *Helicobacter pylori* infection in prostate tumors. Curr Urol.

[46] Glenn W.K., Ngan C.C., Amos T.G. (2017). High risk human *Papilloma* viruses (HPVs) are present in benign prostate tissues before development of HPV associated prostate cancer. Infect Agents Cancer.

[47] Keller E.X., Delbue S., Tognon M., Provenzano M. (2015). Polyomavirus BK and prostate cancer: a complex interaction of potential clinical relevance. Rev Med Virol.

[48] Taghavi A., Mohammadi-Torbati P., Kashi A.H., Rezaee H., Vaezjalali M. (2015). Polyomavirus hominis 1(BK virus) infection in prostatic tissues: cancer versus hyperplasia. Urol J.

[49] Miyake M., Ohnishi K., Hori S. (2019). *Mycoplasma genitalium* infection and chronic inflammation in human prostate cancer: detection using prostatectomy and needle biopsy specimens. Cells.

[50] Wei M.Y., Shi S., Liang C. (2019). The microbiota and microbiome in pancreatic cancer: more influential than expected. Mol Cancer.

[51] Dickson I. (2018). Microbiome promotes pancreatic cancer. Nat Rev Gastroenterol Hepatol.

[52] Geller L.T., Barzily-Rokni M., Danino T. (2017). Potential role of intratumor bacteria in mediating tumor resistance to the chemotherapeutic drug gemcitabine. Science.

[53] Mitsuhashi K., Nosho K., Sukawa Y. (2015). Association of *Fusobacterium* species in pancreatic cancer tissues with molecular features and prognosis. Oncotarget.

[54] Riquelme E., Zhang Y., Zhang L. (2019). Tumor microbiome diversity and composition influence pancreatic cancer outcomes. Cell.

[55] Jin Y., Gao H., Chen H. (2013). Identification and impact of hepatitis B virus DNA and antigens in pancreatic cancer tissues and adjacent non-cancerous tissues. Cancer Lett.

[56] Aykut B., Pushalkar S., Chen R. (2019). The fungal mycobiome promotes pancreatic oncogenesis via activation of MBL. Nature.

[57] Kiss B., Mikó E., Sebő É. (2020). Oncobiosis and microbial metabolite signaling in pancreatic adenocarcinoma. Cancers.

[58] Marshall B.J., Warren J.R. (1984). Unidentified curved bacilli in the stomach of patients with gastritis and peptic ulceration. Lancet.

[59] Chen X.H., Wang A., Chu A.N., Gong Y.H., Yuan Y. (2019). Mucosa-associated microbiota in gastric cancer tissues compared with non-cancer tissues. Front Microbiol.

[60] Delgado S., Cabrera-Rubio R., Mira A., Suárez A., Mayo B. (2013). Microbiological survey of the human gastric ecosystem using culturing and pyrosequencing methods. Microb Ecol.

[61] Eun C.S., Kim B.K., Han D.S. (2014). Differences in gastric mucosal microbiota profiling in patients with chronic gastritis, intestinal metaplasia, and gastric cancer using pyrosequencing methods. Helicobacter.

[62] Yang J., Zhou X., Liu X., Ling Z., Ji F. (2021). Role of the gastric microbiome in gastric cancer: from carcinogenesis to treatment. Front Microbiol.

[63] Dicksved J., Lindberg M., Rosenquist M., Enroth H., Jansson J.K., Engstrand L. (2009). Molecular characterization of the stomach microbiota in patients with gastric cancer and in controls. J Med Microbiol.

[64] Liu X., Shao L., Liu X. (2019). Alterations of gastric mucosal microbiota across different stomach microhabitats in a cohort of 276 patients with gastric cancer. EBioMedicine.

[65] Wang L., Zhou J., Xin Y. (2016). Bacterial overgrowth and diversification of microbiota in gastric cancer. Eur J Gastroenterol Hepatol.

[66] Aviles-Jimenez F., Vazquez-Jimenez F., Medrano-Guzman R., Mantilla A., Torres J. (2014). Stomach microbiota composition varies between patients with non-atrophic gastritis and patients with intestinal type of gastric cancer. Sci Rep.

[67] Rajilic-Stojanovic M., Figueiredo C., Smet A. (2020). Systematic review: gastric microbiota in health and disease. Aliment Pharmacol Ther.

[68] Cantalupo P.G., Katz J.P., Pipas J.M. (2018). Viral sequences in human cancer. Virology.

[69] Shibata D., Weiss L.M. (1992). Epstein-Barr virus-associated gastric adenocarcinoma. Am J Pathol.

[70] Zhao Y., Zhang J., Cheng A.S.L., Yu J., To K.F., Kang W. (2020). Gastric cancer: genome damaged by bugs. Oncogene.

[71] Papon N., Hohl T.M., Zhai B. (2021). Mycobiota dysbiosis and gastric tumorigenesis. Theranostics.

[72] Omar Al-Hassi H., Ng O., Brookes M. (2018). Tumour-associated and non-tumour-associated microbiota in colorectal cancer. Gut.

[73] Gao R., Kong C., Huang L. (2017). Mucosa-associated microbiota signature in colorectal cancer. Eur J Clin Microbiol Infect Dis.

[74] Gao Z., Guo B., Gao R., Zhu Q., Qin H. (2015). Microbiota disbiosis is associated with colorectal cancer. Front Microbiol.

[75] Burns M.B., Lynch J., Starr T.K., Knights D., Blekhman R. (2015). Virulence genes are a signature of the microbiome in the colorectal tumor microenvironment. Genome Med.

[76] Phipps O., Quraishi M.N., Dickson E.A. (2021). Differences in the on- and off-tumor microbiota between right- and left-sided colorectal cancer. Microorganisms.

[77] Chen W., Liu F., Ling Z., Tong X., Xiang C. (2012). Human intestinal lumen and mucosa-associated microbiota in patients with colorectal cancer. PLoS One.

[78] Ramos A., Hemann M.T. (2017). Drugs, bugs, and cancer: *fusobacterium nucleatum* promotes chemoresistance in colorectal cancer. Cell.

[79] Lee D.W., Han S.W., Kang J.K. (2018). Association between *Fusobacterium nucleatum*, pathway mutation, and patient prognosis in colorectal cancer. Ann Surg Oncol.

[80] Mima K., Nishihara R., Qian Z.R. (2016). *Fusobacterium nucleatum* in colorectal carcinoma tissue and patient prognosis. Gut.

[81] Izi S., Youssefi M., Mohammadian Roshan N., Azimian A., Amel Jamehdar S., Zahedi Avval F. (2021). Higher detection of JC polyomavirus in colorectal cancerous tissue after pretreatment with topoisomerase I enzyme; colorectal tissue serves as a JCPyV persistence site. Exp Mol Pathol.

[82] Nagi K., Gupta I., Jurdi N. (2021). Copresence of high-risk human papillomaviruses and epstein-barr virus in colorectal cancer: a tissue microarray and molecular study from Lebanon. Int J Mol Sci.

[83] Li Y., Qiu Q., Fan Z., He P., Chen H., Jiao X. (2018). Th17 cytokine profiling of colorectal cancer patients with or without enterovirus 71 antigen expression. Cytokine.

[84] Mira-Pascual L., Cabrera-Rubio R., Ocon S. (2015). Microbial mucosal colonic shifts associated with the development of colorectal cancer reveal the presence of different bacterial and archaeal biomarkers. J Gastroenterol.

[85] Sipos A., Ujlaki G., Mikó E. (2021). The role of the microbiome in ovarian cancer: mechanistic insights into oncobiosis and to bacterial metabolite signaling. Mol Med.

[86] Shanmughapriya S., SenthilKumar G., Vinodhini K., Das B.C., Vasanthi N., Natarajaseenivasan K. (2012). Viral and bacterial aetiologies of epithelial ovarian cancer. Eur J Clin Microbiol Infect Dis.

[87] Xu J., Peng J.J., Yang W., Fu K., Zhang Y. (2020). Vaginal microbiomes and ovarian cancer: a review. Am J Cancer Res.

[88] Paradowska E., Jabłońska A., Studzińska M., Wilczyński M., Wilczyński J.R. (2019). Detection and genotyping of CMV and HPV in tumors and fallopian tubes from epithelial ovarian cancer patients. Sci Rep.

[89] Cox M., Kartikasari A.E.R., Gorry P.R., Flanagan K.L., Plebanski M. (2021). Potential impact of human cytomegalovirus infection on immunity to ovarian tumours and cancer progression. Biomedicines.

[90] Gonzalez-Bosquet J., Pedra-Nobre S., Devor E.J. (2021). Bacterial, Archaea, and viral transcripts (BAVT) expression in gynecological cancers and correlation with regulatory regions of the genome. Cancers (Basel).

[91] Poore G.D., Kopylova E., Zhu Q. (2020). Microbiome analyses of blood and tissues suggest cancer diagnostic approach. Nature.

[92] Sun L., Ke X., Guan A. (2023). Intratumoural microbiome can predict the prognosis of hepatocellular carcinoma after surgery. Clin Transl Med.

[93] Yamamura K., Baba Y., Nakagawa S. (2016). Human microbiome *Fusobacterium nucleatum* in esophageal cancer tissue is associated with prognosis. Clin Cancer Res.

[94] Wong-Rolle A., Wei H.K., Zhao C., Jin C. (2021). Unexpected guests in the tumor microenvironment: microbiome in cancer. Protein Cell.

[95] Yang L., Li A., Wang Y., Zhang Y. (2023). Intratumoral microbiota: roles in cancer initiation, development and therapeutic efficacy. Signal Transduct Targeted Ther.

[96] Fu A., Yao B., Dong T., Cai S. (2023). Emerging roles of intratumor microbiota in cancer metastasis. Trends Cell Biol.

[97] Narunsky-Haziza L., Sepich-Poore G.D., Livyatan I. (2022). Pan-cancer analyses reveal cancer-type-specific fungal ecologies and bacteriome interactions. Cell.

[98] Xue C., Jia J., Gu X. (2022). Intratumoral bacteria interact with metabolites and genetic alterations in hepatocellular carcinoma. Signal Transduct Targeted Ther.

[99] Guo S., Chen J., Chen F., Zeng Q., Liu W.L., Zhang G. (2020). Exosomes derived from *Fusobacterium nucleatum*-infected colorectal cancer cells facilitate tumour metastasis by selectively carrying miR-1246/92b-3p/27a-3p and CXCL16. Gut.

[100] Lee J.E., Kim H.J., Bae J.Y. (2005). Neogenin expression may be inversely correlated to the tumorigenicity of human breast cancer. BMC Cancer.

[101] Zhang Q., Liang F., Ke Y. (2015). Overexpression of neogenin inhibits cell proliferation and induces apoptosis in human MDA-MB-231 breast carcinoma cells. Oncol Rep.

[102] Garrett W.S. (2015). Cancer and the microbiota. Science.

[103] Krishnamurthy N., Kurzrock R. (2018). Targeting the Wnt/beta-catenin pathway in cancer: update on effectors and inhibitors. Cancer Treat Rev.

[104] Rubinstein M.R., Baik J.E., Lagana S.M. (2019). *Fusobacterium nucleatum* promotes colorectal cancer by inducing Wnt/β-catenin modulator Annexin A1. EMBO Rep.

[105] Wu D., Qiu Y., Jiao Y., Qiu Z., Liu D. (2020). Small molecules targeting HATs, HDACs, and BRDs in cancer therapy. Front Oncol.

[106] Tzeng A., Sangwan N., Jia M. (2021). Human breast microbiome correlates with prognostic features and immunological signatures in breast cancer. Genome Med.

[107] Kovács T., Mikó E., Vida A. (2019). Cadaverine, a metabolite of the microbiome, reduces breast cancer aggressiveness through trace amino acid receptors. Sci Rep.

[108] Tang W., Putluri V., Ambati C.R., Dorsey T.H., Putluri N., Ambs S. (2019). Liver- and microbiome-derived bile acids accumulate in human breast tumors and inhibit growth and improve patient survival. Clin Cancer Res.

[109] Gur C., Ibrahim Y., Isaacson B. (2015). Binding of the Fap2 protein of *Fusobacterium nucleatum* to human inhibitory receptor TIGIT protects tumors from immune cell attack. Immunity.

[110] Mima K., Sukawa Y., Nishihara R. (2015). *Fusobacterium nucleatum* and T cells in colorectal carcinoma. JAMA Oncol.

[111] Kalaora S., Nagler A., Nejman D. (2021). Identification of bacteria-derived HLA-bound peptides in melanoma. Nature.

[112] Ravnik Z., Muthiah I., Dhanaraj P. (2021). Computational studies on bacterial secondary metabolites against breast cancer. J Biomol Struct Dyn.

[113] Liu F., Li J., Guan Y. (2019). Dysbiosis of the gut microbiome is associated with tumor biomarkers in lung cancer. Int J Biol Sci.

[114] Ren Z., Li A., Jiang J. (2019). Gut microbiome analysis as a tool towards targeted non-invasive biomarkers for early hepatocellular carcinoma. Gut.

[115] Zhou Z., Ge S., Li Y. (2020). Human gut microbiome-based knowledgebase as a biomarker screening tool to improve the predicted probability for colorectal cancer. Front Microbiol.

[116] Temraz S., Nassar F., Nasr R., Charafeddine M., Mukherji D., Shamseddine A. (2019). Gut microbiome: a promising biomarker for immunotherapy in colorectal cancer. Int J Mol Sci.

[117] Wong S.H., Kwong T.N.Y., Chow T.C. (2017). Quantitation of faecal *Fusobacterium* improves faecal immunochemical test in detecting advanced colorectal neoplasia. Gut.

[118] Shen X., Li J., Li J. (2021). Fecal *Enterotoxigenic Bacteroides fragilis-Peptostreptococcus stomatis-Parvimonas micra* biomarker for noninvasive diagnosis and prognosis of colorectal laterally spreading tumor. Front Oncol.

[119] Huang S.T., Chen J., Lian L.Y. (2020). Intratumoral levels and prognostic significance of *Fusobacterium nucleatum* in cervical carcinoma. Aging (Albany NY).

[120] Yamamura K., Izumi D., Kandimalla R. (2019). Intratumoral *Fusobacterium nucleatum* levels predict therapeutic response to neoadjuvant chemotherapy in esophageal squamous cell carcinoma. Clin Cancer Res.

[121] Yu T., Guo F., Yu Y. (2017). *Fusobacterium nucleatum* promotes chemoresistance to colorectal cancer by modulating autophagy. Cell.

[122] Peng R., Liu S., You W. (2022). Gastric microbiome alterations are associated with decreased CD8^+^ tissue-resident memory T cells in the tumor microenvironment of gastric cancer. Cancer Immunol Res.

[123] Ji H., Jiang Z., Wei C. (2023). Intratumoural microbiota: from theory to clinical application. Cell Commun Signal.

[124] Colbert L.E., El Alam M.B., Wang R. (2023). Tumor-resident *Lactobacillus iners* confer chemoradiation resistance through lactate-induced metabolic rewiring. Cancer Cell.

[125] Xuan Y., Wang H., Yung M.M. (2022). SCD1/FADS2 fatty acid desaturases equipoise lipid metabolic activity and redox-driven ferroptosis in ascites-derived ovarian cancer cells. Theranostics.

[126] Bender M.J., McPherson A.C., Phelps C.M. (2023). Dietary tryptophan metabolite released by intratumoral *Lactobacillus reuteri* facilitates immune checkpoint inhibitor treatment. Cell.

[127] Du Y., Liu L., Ma W. (2023). The role of extratumoral and intratumoral microorganisms in cancer immunotherapy. Innov Life.

[128] Shi Y., Zheng W., Yang K. (2020). Intratumoral accumulation of gut microbiota facilitates CD47-based immunotherapy via STING signaling. J Exp Med.

[129] Sivan A., Corrales L., Hubert N. (2015). Commensal *Bifidobacterium* promotes antitumor immunity and facilitates anti-PD-L1 efficacy. Science.

[130] Michaud D.S., Izard J., Wilhelm-Benartzi C.S. (2013). Plasma antibodies to oral bacteria and risk of pancreatic cancer in a large European prospective cohort study. Gut.

[131] Del Castillo E., Meier R., Chung M. (2019). The microbiomes of pancreatic and duodenum tissue overlap and are highly subject specific but differ between pancreatic cancer and noncancer subjects. Cancer Epidemiol Biomarkers Prev.

[132] Lu S.Y., Hua J., Xu J. (2021). Microorganisms in chemotherapy for pancreatic cancer: an overview of current research and future directions. Int J Biol Sci.

[133] Shiao S.L., Kershaw K.M., Limon J.J. (2021). Commensal bacteria and fungi differentially regulate tumor responses to radiation therapy. Cancer Cell.

[134] Hou J., Zheng H., Li P., Liu H., Zhou H., Yang X. (2018). Distinct shifts in the oral microbiota are associated with the progression and aggravation of mucositis during radiotherapy. Radiother Oncol.

[135] Sevcikova A., Izoldova N., Stevurkova V. (2022). The impact of the microbiome on resistance to cancer treatment with chemotherapeutic agents and immunotherapy. Int J Mol Sci.

[136] Trédan O., Galmarini C.M., Patel K., Tannock I.F. (2007). Drug resistance and the solid tumor microenvironment. J Natl Cancer Inst.

[137] Ma W., Zhang L., Chen W. (2024). Microbiota enterotoxigenic *Bacteroides fragilis*-secreted BFT-1 promotes breast cancer cell stemness and chemoresistance through its functional receptor NOD1. Protein Cell.

[138] Zheng H., Chen X., Li Q., Liu Y., Cai J. (2023). Effects of chemotherapy and immunotherapy on microbial diversity in TME and engineered bacterial-mediated tumor therapy. Front Immunol.

[139] He Y., Liu Q.W., Liao H.X., Xu W.W. (2021). Microbiota in cancer chemoradiotherapy resistance. Clin Transl Med.

[140] Kong F., Fang C., Zhang Y. (2022). Abundance and metabolism disruptions of intratumoral microbiota by chemical and physical actions unfreeze tumor treatment resistance. Adv Sci (Weinh).

[141] Wang J.W., Chen Q.W., Luo G.F. (2021). A self-driven bioreactor based on bacterium-metal-organic framework biohybrids for boosting chemotherapy via cyclic lactate catabolism. ACS Nano.

[142] Deng Y., Hou X., Wang H., Du H., Liu Y. (2024). Influence of gut microbiota-mediated immune regulation on response to chemotherapy. Pharmaceuticals.

[143] Omori M., Kato-Kogoe N., Sakaguchi S. (2023). Characterization of oral microbiota following chemotherapy in patients with hematopoietic malignancies. Integr Cancer Ther.

[144] Chiba A., Bawaneh A., Velazquez C. (2020). Neoadjuvant chemotherapy shifts breast tumor microbiota populations to regulate drug responsiveness and the development of metastasis. Mol Cancer Res.

[145] Gurbatri C.R., Lia I., Vincent R. (2020). Engineered probiotics for local tumor delivery of checkpoint blockade nanobodies. Sci Transl Med.

[146] Song W., Anselmo A.C., Huang L. (2019). Nanotechnology intervention of the microbiome for cancer therapy. Nat Nanotechnol.

[147] Liu S., Xu X., Zeng X., Li L., Chen Q., Li J. (2014). Tumor-targeting bacterial therapy: a potential treatment for oral cancer (Review). Oncol Lett.

[148] Flickinger JC Jr, Rodeck U., Snook A.E. (2018). *Listeria monocytogenes* as a vector for cancer immunotherapy: current understanding and progress. Vaccines (Basel).

[149] Kubiak A.M., Minton N.P. (2015). The potential of clostridial spores as therapeutic delivery vehicles in tumour therapy. Res Microbiol.

[150] Chowdhury S., Castro S., Coker C., Hinchliffe T.E., Arpaia N., Danino T. (2019). Programmable bacteria induce durable tumor regression and systemic antitumor immunity. Nat Med.

[151] Canale F.P., Basso C., Antonini G. (2021). Metabolic modulation of tumours with engineered bacteria for immunotherapy. Nature.

[152] Schmitz-Winnenthal F.H., Hohmann N., Schmidt T. (2018). A phase 1 trial extension to assess immunologic efficacy and safety of prime-boost vaccination with VXM01, an oral T cell vaccine against VEGFR2, in patients with advanced pancreatic cancer. OncoImmunology.

[153] Yoon W., Yoo Y., Chae Y.S., Kee S.H., Kim B.M. (2018). Therapeutic advantage of genetically engineered *Salmonella typhimurium* carrying short hairpin RNA against inhibin alpha subunit in cancer treatment. Ann Oncol.

[154] Redelman-Sidi G., Glickman M.S., Bochner B.H. (2014). The mechanism of action of BCG therapy for bladder cancer: a current perspective. Nat Rev Urol.

