# Supplementary material for: The specific hallmarks, emerging roles, key mechanisms, and clinical applications of intra-tumoral microbiota in human cancers
Source: Genes Dis. 2025 Jun 22;13(1):101733. doi: 10.1016/j.gendis.2025.101733 (PMC12505679; doi:10.1016/j.gendis.2025.101733)
Supplement: Multimedia component 1 [file mmc1.docx]

**Table S1** An overview of intra-tumoral microbiota associated with different cancers with the detailed information of microbiota, and the phylum, class and order to which these microbes belong.

| **Cancer types** | **Subtype** | **Differential Bacterial signatures** | **Differential Viral signatures** | **Differential Fungal signatures** | **Differential Parasitic signatures** | **Differential Archaeal signatures** |
| --- | --- | --- | --- | --- | --- | --- |
| Breast cancer | Breast cancer | Family: Enterobacteriaceae↓, Micrococcaceae↑, Caulobacteraceae↑, Rhodobacteraceae↑, Nocardioidaceae↑, Methylobacteriaceae ↑, Bacteroidaceae↓, Bartonella, Coxiella | Family: Adenoviridae, Anelloviridae, Arenaviridae, Bunyaviridae, Coronaviridae, Filoviridae, Flaviviridae, Herpesviridae, Iridoviridae, Papillomaviridae, Paramyxoviridae, Parvoviridae, Picornaviridae, *Poxviridae*, Reoviridae, Retroviridae, Rhabdoviridae | Genus: Ajellomyces↑, Alternaria↑, Cunninghamella↑, Epidermophyton↑, Filobasidiella↑, Rhizomucor↑, Trichophyton↑ | N/A | N/A |
|  |  | Phylum: Proteobacteria, Firmicutes, Actinobacteria, Bacteroidetes |  | Species: Pleistophora mulleris, Piedraia hortae, Paecilomyces reniformis, Phialophora verrucosa, Fonsecaea pedrosoi | N/A | N/A |
|  |  | Genus: Bacillus, Enterobacteriaceae↑, Staphylococcus↑, Lactococccus↓, Streptococcus↓, Fusobacterium↑, Atopobium↑, Gluconacetobacter↑, Hydrogenophaga↑, Lactobacillus↑, Actinomyces, Brevundimonas, Mobiluncus, Mycobacterium, Sphingomona, Propionicimonas↑, Agrococcus↑ |  | N/A |  |  |
|  | ER- | Family: Actinomycetaceae↑ | N/A | N/A | N/A | N/A |
|  |  | Genus: Streptophyta_UF_UG116↑, Alkanindiges↑, Lautropia_US38 ↑, Sphingomonas_US124↑ |  |  |  |  |
|  |  | Species: Actinomyces odontolyticus↑ |  |  |  |  |
|  | ER+ | Genus: Arcanobacterium↑, Bifidobacterium↑, Cardiobacterium↑, Citrobacter↑, Escherichia↑, Bartonella↑ | N/A | Genus: Filobasidiella, Mucor, Trichophyton | Genus: Brugia, Paragonimus | N/A |
|  | HER2+ | Genus: Granulicatella_US31↑, Streptococcus↑, Dyadobacter↑ | Family: Nodaviridae | Genus: Epidermophyton, Fonsecaea, Pseudallescheria | Genus: Balamuthia | N/A |
|  | TPBC | Genus: Bordetella↑, Campylobacter↑, Legionella↑, Pasteurella↑ | Family: Birnaviridae, Hepeviridae | Genus: Penicillium | Genus: Ancylostoma↑, Angiostrongylus↑, Echinococcus↑, Sarcocystis↑, Trichomonas↑, Trichostrongylus ↑ | N/A |
|  | TNBC | Family: Nocardiopsaceae↑ | Family: Herpesviridae, Retroviridae, Parapoxviridae, Polyomaviridae, Papillomaviridae | Genus: Alternaria, Malassezia, Piedraia, Rhizomucor | Genus: Centrocestus, Contracaecum, Leishmania, Necator, Onchocerca, Toxocara, Trichinella,Trichuris, Mansonella, Strongyloides | N/A |
|  |  | Genus: Aerococcus↑, Arcobacter↑, Geobacillus↑, Orientia↑, Rothia↑, Bacillus_US21↑, Leptotrichia_US21↑, Streptophyta_UF_UG116↑, Turicibacter ↑, Achromobacter↑, Actinomyces↓ | Species: Human cytomegalovirus, Human herpes virus 1, Human herpes virus-8, Epstein Barr virus, Fujinami Sarcoma virus, Mouse mammary tumor virus, Merkel cell Polyomavirus, Simian Virus 40, Human papilloma virus (HPV) 6b, HPV18, HPV2, HPV16 | Species: Pleistophora mulleris, Piedraia hortae, Paecilomyces reniformis, Phialophora verrucosa, Fonsecaea pedrosoi | Species: Trichuris trichura, Thelazia gulosa, Leishmania |  |
|  |  | Species: Achromobacter denitrificans↑ |  |  |  |  |
| Lung cancer | | Phylum: Proteobacteria↑, Firmicutes↓ | *Trichodysplasia spinulosa-associated polyomavirus* | N/A | N/A | N/A |
|  |  | Family: Lachnospiraceae↓, Ruminococcaceae↓,  Bacteroidaceae↓ |  |  |  |  |
|  |  | Genus: Thermus↑, Legionella↑, Propionibacterium ↓, *Streptococcus*↑, Staphylococcus↓,  *Dialister*↓, Neisseria↑,  *Bacteroides*↓,  *Ruminococcus*↓,  *Roseburia*↓,  Faecalibacterium↑,  *Sphingomonadaceae*↑,  Escherichia-Shigella ↑, Alloprevotella↑, Pseudomonas↑, Brevundimonas↑, Koribacteraceae ↑ |  |  |  |  |
| Prostate cancer | | Phylum: Proteobacteria, Firmicutes, Actinobacteria, Bacteroidetes | Species: HPV16, *HPV18*, *Epstein Barr virus*, *Human cytomegalovirus*, *JC polyomavirus*↑, *BK polyomavirus*↑ | Phylum: *Ascomycetes* | Phylum: Nematoda | N/A |
|  |  | Genus: *Propionibacterium spp.*↑, *Staphylococcus spp.*↑, *Pseudomonas*↑, *Escherichia*↑, Acinetobacter↑, Propionibacterium↑ | Family: Alpharetrovirus RSV, Betaretrovirus MMTV, Gammaretrovirus MMLV | Family: Dermatophytes, Yeasts, Zygomycetes, Microsporidia | Classis: Sarcomastigophora, Phylum: Platyhelminthes, Apicomplexa, Acanthocephala |  |
|  |  | Species: *Propionibacterium acnes*, *Helicobacter pylori* |  |  | Species: Chlamydia trachomatis, Mycoplasma genitalium |  |
| Pancreatic cancer | | Phylum: *Proteobacteria*↑, *Bacteroidetes*↑, *Firmicutes*↑, | *Hepatitis B virus* | Genus: Malassezia | N/A | N/A |
|  | | Genus: *Bifidobacteria*↑, *Gammaproteobacteria*↑, *Helicobacter pylori*, *Clostridium*↑, *Fusobacterium* |  |  |  |  |
|  |  | Species: *Porphyromonas gingivalis*↑, *Sachharopolyspora*↑, Pseudoxanthomonas↑, *Streptomyces*↑, *Bacillus clausii*↑ |  |  |  |  |
| Gastric cancer | | Phylum: Proteobacteria↓, Firmicutes↑, Bacteroidetes, Actinobacteria↑, Acidobacteria↑, Fusobacteria↑, Candidatus Saccharibacteria (TM7)↓ | Species: Epstein Barr virus, Cytomegalovirus, Human herpes virus 6 | *Alternaria*↑, Candida ↑, Thermomyces↓, Saitozyma↓ | N/A | N/A |
|  |  | Genus: Peptostreptococcus↑, Streptococcus↑, Fusobacterium↑, Lactobacillus, Lactococcus, Veillonella, Prevotella, Pseudomonas↑, Bacilli, Porphyromonas spp↓, Neisseria spp↓, Haemophilus |  |  |  |  |
|  |  | Species: Helicobacter pylori↓, Prevotella copri↓, Bacteroides uniformis↓, Prevotella melaninogenica↑, Streptococcus anginosus↑, Streptococcus sinensis↓, Propionibacterium acnes↑, Lactobacillus coleohominis↑ |  |  |  |  |
|  |  | Family: *Lachnospiraceae*↑, *Helicobacteraceae*↓ |  |  |  |  |
| Colorectal cancer | | Phylum: Bacteroidetes↑, Firmicutes↑, Fusobacteria↑ | *Enteroviruses 71*, JC polyomavirus, Human papilloma viruses (16, 18, 31, 33, 35, 45, 51, 52, and 58), Epstein Barr virus | N/A | N/A | *Methanobacteriales* |
|  |  | Genus: Lactobacillales↑, Lactococcus↑, *Bacteroides* ↑, Fusobacterium↑, Faecalibacterium↓, Pseudomonas↓, Escherichia-Shigella↓ |  |  |  | *Methanobrevibacterium*, Methanobrevibacter smithii |
|  |  | Species: Fusobacterium nucleatum ↑ |  |  |  |  |
| Ovarian cancer | | Genus: Pediococcus↑, Sphingomonas↑, Chryseobacterium↑, Enterococcus↑, Staphylococcus↑, Treponema↑, Francisella↑, Shewanella↑, Brucella↑, Chlamydia↑, *Mycoplasma*↑ | Family: Poxviridae, Retroviridae, Polyomaviridae | Genus: Cladosporium, Pneumocystis, Acremonium, Cladophialophora, Malassezia | *Chlamydia*, Dipylidium, Trichuris, Leishmania | N/A |
|  |  |  | Species: Human Papillomavirus (16, 18, 2, 4, 5, 6b, 7, 10, 32, 48, 49, 50, 60, 54, 92, 96, 101, 128, 129, 131, 132), Cytomegalovirus | Species: Microsporidia Pleistophora |  |  |
| Other cancers | Melanoma | Clostridium, Gardnerella vaginalis | N/A | N/A | N/A | N/A |
|  | Oral squamous cell carcinoma | *Fusobacterium*, *Treponema* | N/A |  |  |  |
|  | Liver cancer | Phylum: *Actinobacteria*, *Proteobacteria*  *Firmicutes* | *Hepatitis B virus*, Hepatitis C virus |  |  |  |
|  | Esophageal cancer | *Fusobacterium nucleatum* | N/A |  |  |  |
|  | Bone cancer | *Actinomycetes*↑, Lactobacillales↑ | N/A |  |  |  |
|  | Glioblastoma multiforme | Phylum: Proteobacteria | N/A |  |  |  |

↑Represents microbiota enriched in cancer compared to controls. ↓represents microbiota decreased in cancer compared to controls.
